# Supplementary material for: Decentralized trust optimization in VANETs: A blockchain-driven hybrid PoS-PBFT architecture for enhanced security and energy-efficient communication
Source: PLoS One. 2025 Dec 15;20(12):e0338618. doi: 10.1371/journal.pone.0338618 (PMC12704869; doi:10.1371/journal.pone.0338618)
Supplement: Attachment — Submitted filename: Response to Reviewers Rebuttal letter.pdf [file pone.0338618.s001.pdf]

Manuscript ID: PONE-D-25-29277

Original Article Title: "Decentralized Trust Optimization in VANETs: A Blockchain-Driven Hybrid PoS-PBFT Architecture for Enhanced Security and Energy-Efficient Communication"

Dear Editor,

Thank you for allowing a resubmission of our manuscript, with an opportunity to address the reviewers' comments. The revised draft of our Manuscript ID **PONE-D-25-29277entitled "Decentralized Trust Optimization in VANETs: A Blockchain-Driven Hybrid PoS-PBFT Architecture for Enhanced Security and Energy-Efficient Communication"** to **PLOS One**. We are thankful to you for spending your precious time thoroughly reviewing our manuscript in light of the remarks given by two worthy reviewers. We are uploading (a) our point-by-point response to the comments (below) (response to reviewers), (b) an updated manuscript with yellow highlighting indicating changes (*Supplementary Material for Review*), and (c) a clean updated manuscript without highlights (*Main Manuscript)*.

Best Regards

Asad Ullah et al.

Reviewer #1: Comments to the Author

The paper describes an infrastructure to provide trust in VANETs. The proposal is technically sound in general terms. The claims in the introduction are justified, and the problem is correctly described. Descriptions are OK. Figures, mathematical expressions and code are provided. I think this is enough to ensure future replicability and readability.

References are timely, and the topic matches the scope of the journal.

Results are also coherent with the state of the art and validate the initial hypotheses. However, in my opinion, some improvements need to be made to this section:

Reviewer#1, Concern # 1: First, you need to describe your experimental setup in detail. Software and hardware you used. How the results were calculated. Did you consider numerical problems and errors? How did you manage the experimental biases and errors?

Author Response:

We sincerely thank the esteemed reviewer for highlighting the point regarding the clarity and transparency of our experimental setup.

We have added a new section (Section 5 Experimental Setup and Evaluation Protocol) that describes in detail the experimental setup, including software and hardware specifications,

simulation environment, data generation process, error handling strategies, and bias mitigation techniques.

Author Action:

In the updated manuscript, the changes with YELLOW color in section 5 are highlighted**.** **"Experimental Setup and Evaluation Protocol"** in the revised manuscript.

5 Experimental Setup and Evaluation Protocol

5.1 Simulation Tools and Environment

This section presents the experimental framework adopted to validate the proposed decentralized trust management mechanism for VANETs. The proposed trust management framework is designed primarily for small- to medium-scale VANET scenarios such as urban zones or localized vehicular regions. The simulation settings were chosen to allow controlled experimentation and repeatability while capturing the trust dynamics in decentralized environments. Large-scale deployment and real-world testing are identified as future work directions due to the infrastructural and cost constraints involved.

To evaluate the system under realistic traffic and communication dynamics, an integrated co-simulation setup is employed. Vehicular mobility was modelled using the Simulation of Urban MObility (SUMO), while message dissemination and protocol simulation were handled by OMNeT++ integrated through the Veins framework [54]. The trust evaluation logic, including the Gaussian Naive Bayes (GaussianNB) model and blockchain mechanisms, was implemented in Python 3.11. For distributed ledger validation, a lightweight prototype was deployed using Hyperledger Fabric v2.4. Scikit-learn was used for training and testing the machine learning classifiers.

5.2 Simulation Scenario and Parameters

The proposed approach was evaluated in a simulated VANET environment consisting of 100 vehicles dispersed across an area of 1 square kilometer. Vehicles moved with speeds ranging from 10 to 20 m/s and operated under a communication radius of 300 meters. Of these, 20 vehicles were configured as malicious actors, while 80 operated legitimately. Simulations captured trust value fluctuations for both legitimate and malicious vehicles. The effectiveness of the trust management framework was quantified using the detection rate of malicious nodes and compared against benchmark approaches. Additionally, the performance of the V2V authentication mechanism was examined in terms of computational overhead, and the proposed hybrid consensus algorithm was evaluated relative to conventional alternatives. The simulation results provided a quantitative basis for asserting the superiority of the proposed trust mechanism in terms of accuracy, efficiency, and resistance to malicious manipulation [55, 56].

Algorithm 2 Algorithm for Hybrid Consensus Mechanism (PoS + PBFT) for Trust Management in VANETs

Require:
Set of vehicles $V = \{v_1, v_2, \ldots, v_n\}$ with trust stakes $S(v_1), S(v_2), \ldots, S(v_n)$
Number of validators required n_v
Fault tolerance parameter f (maximum faulty nodes tolerated)

Ensure:
Consensus on trust scores for vehicle messages, $T_f(v_i)$, and commitment to blockchain

procedure HybridConsensus(V, n_v, f)
  Phase 1: Validator Selection via Proof of Stake (PoS)
  Calculate the total stake $S_{total} = \sum_{i=1}^{n} S(v_i)$
  for all vehicles $v_i \in V$ **do**
    Compute selection probability for v_i: $P(v_i) = \frac{S(v_i)}{S_{total}}$
  end for
  Select n_v validators randomly based on $P(v_i)$
  {Validators are now selected and ready for PBFT consensus}
  Phase 2: Pre-Prepare (PBFT)
  Let the primary validator v_p propose a block B with trust scores $T_s(v_1), T_s(v_2), \ldots, T_s(v_n)$
  for all validators v_i **do**
    Receive proposed block B from v_p
    {Validators review the block for trust scores}
  end for
  Phase 3: Prepare (PBFT)
  Each validator verifies trust scores $T_s(v_i)$ for each vehicle based on local observations
  for all validators v_i **do**
    if validator agrees with trust scores in B **then**
      Broadcast vote $V_i(B) = 1$ {Vote in favor of the block}
    else
      Broadcast vote $V_i(B) = 0$ {Vote against the block}
    end if
  end for
  Phase 4: Commit (PBFT)
  Count the total votes: $V_{total} = \sum_{i=1}^{n_v} V_i(B)$
  if $V_{total} \geq 2f + 1$ **then**
    {Supermajority achieved, commit block to blockchain}
    Commit the trust scores $T_f(v_i)$ for each vehicle to the blockchain
    Broadcast the committed block B to all nodes
  else
    {Consensus failed, block is discarded}
    Reject the block and restart the consensus process
  end if
  Phase 5: Finalization and Block Propagation
  Propagate the committed block across the network
  Update trust scores of vehicles based on committed block B
end procedure
=0

Table 5. Hardware and Software Configuration

Component	Specification
Processor	Intel Core i7-12700K, 12-Core (3.6 GHz)
RAM	32 GB DDR5
Storage	1 TB NVMe SSD
Operating System	Ubuntu 22.04 LTS
Blockchain Platform	Hyperledger Fabric v2.4 (Dockerized)
Simulation Framework	SUMO 1.18, OMNeT++ 6.0, Veins 5.1
Programming Environment	Python 3.11, Scikit-learn 1.3

To emulate a distributed and decentralized environment, multiple validator nodes were instantiated using Docker containers configured with realistic latency and packet loss constraints. 850

5.4 Trust Score Computation and Consensus

Trust scores were computed using the hybrid framework detailed in Section 3. Direct trust was estimated using an Exponential Moving Average (EMA) model, while indirect trust was calculated using a weighted reputation scheme. These trust values were validated through a hybrid PoS and PBFT consensus mechanism, ensuring fault tolerance and resistance to Sybil attacks. Only transactions endorsed by consensus participants were committed to the blockchain ledger, maintaining consistency and transparency in trust propagation.

5.5 Evaluation Metrics and Result Computation

System performance was evaluated using five primary metrics: (i) trust score accuracy, (ii) malicious vehicle detection rate, (iii) false positive rate, (iv) communication overhead, and (v) trust verification latency. Trust accuracy was validated against ground-truth behavioral classifications. Detection and false positives were analyzed through precision-recall statistics, while communication overhead was measured by counting transmitted trust messages per time slot. Latency encompasses the delay from trust computation to blockchain confirmation.

5.6 Numerical Stability and Bias Mitigation

To ensure the numerical reliability of trust computations, all arithmetic operations were performed using 64-bit floating-point precision. Trust scores were normalized within the [0, 1] interval, and adaptive thresholds were used to address rounding errors. Simulations were repeated 100 times using Monte Carlo sampling with varied random seeds to diversify vehicle trajectories and attack patterns. The reported metrics represent the average of these iterations, with 95% confidence intervals. Additionally, k-fold cross-validation was applied to the GaussianNB classifier to avoid overfitting and ensure generalizability.

5.7 Integration with VANET Protocols

The proposed decentralized trust framework is designed as an overlay system that operates on top of standard V2V communication protocols, particularly those based on IEEE 802.11p. The trust-related messages are disseminated via the application layer, while core VANET communication

protocols remain unmodified. This modular design allows compatibility with existing vehicular communication stacks without requiring modifications to lower-layer protocols. Validator selection in the proposed hybrid consensus mechanism is dynamic and mobility-aware. Validator candidates are selected for each consensus epoch based on a weighted combination of their stake history and recent trust stability scores. This strategy ensures that nodes with transient connections are deprioritized, while consistently reliable nodes maintain validator status. A rotating validator committee approach is adopted to handle topological changes without degrading fault tolerance or consensus reliability.

Reviewer#1, Concern # 2: As all your results are based on simulations, you need to discuss the validity threats, at least the internal and external.

Author Response:

We are grateful to the reviewer for this insightful comment. We have incorporated a subsection in the manuscript that discusses the potential threats to validity, focusing on both internal and external factors. This addition strengthens the credibility of our simulation-based evaluation and highlights the precautions taken to ensure the robustness of our findings.

Author Action:

We have updated the manuscript according to the reviewer instructions and highlighted the changes with YELLOW color in **Section 6.10 "Threats to Validity"** in the revised manuscript.

While the simulation-based approach adopted in this study offers a controlled and repeatable environment for evaluating the proposed blockchain-enabled trust management scheme for VANETs, certain validity threats may influence the generalizability and reliability of the results. In this section, the primary threats to both internal and external validity is discussed and describe the mitigation strategies employed.

The Internal Validity refers to the degree to which the observed outcomes can be confidently attributed to the mechanisms and processes embedded within the proposed framework, rather than to extraneous or uncontrolled factors. The simulations after implementation, it is ensured that the internal consistency by initializing all experiments with multiple random seeds to prevent deterministic biases. Each simulation scenario was repeated ten times under varying vehicular densities and mobility speeds, and the average values were reported to smooth out the influence of stochastic variability. Furthermore, trust evaluations were validated using redundant verification procedures to detect outliers and anomalies in the calculated scores. All algorithmic components, including the PoS+PBFT consensus process and trust propagation routines, were independently tested using modular validation scripts before integration into the whole system. Numerical precision and convergence checks were applied where necessary to guard against computational errors. Together, these measures help establish confidence in the integrity and repeatability of the simulation results. External validity refers to the extent to which the results derived from simulations can be generalized to real-world vehicular environments. Although the simulation setup closely mimics practical scenarios in terms of vehicle-to-vehicle (V2V) and vehicle-to-infrastructure (V2I) communication, trust score propagation, and consensus dynamics, certain real-world factors, such as environmental noise, urban signal obstruction, heterogeneous communication hardware, and unpredictable driver behavior were not fully replicated in the

simulation model. The current implementation also assumes that the Trust Authority (TA) and consensus validators operate under ideal network conditions without delays or security breaches, which might not hold in a live deployment. Despite these limitations, the use of parameterizable models and scalable architecture supports future adaptation to more realistic traffic and network conditions. Future work will incorporate co-simulation environments, such as SUMO integrated with NS-3, to account for real traffic flow and communication delays, and ultimately validate the system in testbed or field conditions.

Reviewer#2, Concern # 1: The paper presents a critical contradiction in its core premise. The authors claim to solve "decentralized trust management" while maintaining a centralized Trust Authority (TA) that "is assumed to be completely trustworthy and secure" (Section 3.1.3). This undermines the entire blockchain rationale. If a trusted central authority exists, traditional cryptographic signatures and distributed hash tables would be more efficient than blockchain.

Author Response:

We sincerely thank the reviewer for raising this insightful and important observation. We acknowledge the concern regarding the apparent contradiction between decentralization and the existence of a trusted authority. Our system does not rely on a centralized control model, but instead adopts a hybrid trust architecture where the Trust Authority (TA) performs an auxiliary and supervisory role rather than direct trust computation or validation.

Specifically, the TA is not responsible for executing or approving trust decisions or managing consensus on the blockchain. Instead, it serves as a bootstrapping and governance entity, primarily tasked with initializing the network, issuing initial trust credentials for new nodes, and facilitating the selection of consensus validators. Once initialized, the actual trust evaluation and transaction validation processes are conducted in a fully decentralized manner via the hybrid PoS + PBFT consensus mechanism among distributed validators.

Furthermore, the inclusion of the TA is motivated by practical considerations common in real-world vehicular environments. For instance, transportation authorities or regulatory bodies (e.g., the National Highway Traffic Safety Administration) often require visibility and accountability in safety-critical applications like VANETs. Thus, our model positions the TA as a regulatory oversight body that can track malicious entities and enforce compliance without disrupting the decentralized consensus process.

We have revised subsection 3.1.3 Trust Authority (TA) to emphasize that the TA does not interfere with or replace blockchain consensus, and is instead used to improve system accountability, transparency, and initial trust anchoring, a role similar to the certificate authorities in public-key infrastructures. Moreover, the model maintains resilience against TA compromise since trust scores and validation outcomes remain consensus-driven and decentralized.

Author Action:

We have updated the manuscript according to the reviewer instructions and highlighted the changes with YELLOW color **subsection 3.1.3 Trust Authority (TA)** in the revised manuscript.

The Trust Authority (TA) plays a critical supervisory role in decentralized trust management system. While the TA is assumed to be secure and reliable, as in similar works [1], it does not act as a centralized controller. Instead, it facilitates decentralized trust operations in Vehicular Ad Hoc Networks (VANETs) by overseeing credential issuance, validator selection, and trust bootstrap processes. In the developed architecture, the TA assists in initializing the network by securely registering OBUs and RSUs, issuing cryptographic credentials, and managing the setup of validator nodes. Once initialized, trust evaluations, block generation, and consensus operations are entirely decentralized and carried out through validator nodes using the hybrid PoS+ PBFT mechanism.

Furthermore, the TA maintains visibility over vehicle data and supports real-time identification of potentially malicious entities [50]. It collects the trust scores, T(vi, t), for each vehicle vi over time t, where the scores are derived from both direct and indirect interactions as follows:

$$T(v_i, t) = \alpha \cdot D(v_i, t) + \beta \cdot \sum_{j=1}^{n} w_j R(v_i, v_j, t) \quad (1)$$

Each weight wj is computed as a normalized trust value over recent interactions. Here in Equation 1, D(vi, t) represents the direct trust score based on prior behavior, while R(vi, vj, t) denotes the recommendation of node vj regarding vi. Each recommendation is weighted by wj, reflecting the recommender's trust level. The parameters α and β are weighting coefficients with α + β = 1, and n is the number of neighboring nodes. For newly joined vehicles, the TA computes initial trust values T0(vi) to establish a starting reputation profile.

$$w_j = \frac{T(v_j, t - \Delta t)}{\sum_{k=1}^{n} T(v_k, t - \Delta t)} \quad (2)$$

In Equation 2, the normalization ensures that the collective influence of recommendations remains bounded and reflects current trust standings, allowing the system to gradually converge under stable conditions. As vehicular dynamics shift, trust updates remain responsive yet smooth due to the use of exponentially weighted moving averages (EWMA) in both D(vi, t) and R(vi, vj , t). Importantly, these initializations and tracking processes do not interfere with or influence consensus decisions. All validations and trust adjustments are recorded on-chain and executed via distributed validators. The rationale for incorporating a TA is grounded in practical deployment scenarios, where regulatory or transportation authorities require auditability and accountability in safety-critical networks like VANETs. The TA may also help manage revocations and issuing compliance notices; however, it does not possess decision-making power in the blockchain's consensus or trust updates.

Thus, the Trust Authority in the proposed model serves as a governance and oversight entity that ensures the system remains compliant and secure without compromising decentralization. This design provides a balanced architecture that maintains transparency, scalability, and robustness, while remaining resilient against single points of failure.

Reviewer#2, Concern # 2: The claimed hybrid PoS-PBFT mechanism lacks substantial innovation. The authors merely combine existing consensus algorithms without addressing fundamental compatibility issues:

- PoS requires stake accumulation over time, while VANET nodes are highly mobile
- PBFT requires stable validator sets, incompatible with vehicular mobility patterns
- No analysis of how validator selection transitions occur during high-speed vehicle movement.

Author Response:

We sincerely appreciate the reviewer insightful observations regarding the consensus design. This concern has led us to clarify and significantly expand the explanation of our proposed hybrid PoS–PBFT consensus mechanism in the revised manuscript.

We acknowledge the inherent incompatibility between traditional Proof-of-Stake (PoS) and Practical Byzantine Fault Tolerance (PBFT) when applied directly to highly dynamic environments such as VANETs. In our revised manuscript, we emphasize that our contribution lies not in the raw combination of these protocols but in their contextual adaptation to vehicular networks through three key mechanisms:

(1) **Stake via Trust Accumulation:** Unlike classical PoS, where stake is based on financial assets, we propose a trust-based stake model in which validators are selected based on accumulated behavioral trust scores that reflect long-term reliability and reputation.

(2) **RSU-Assisted Validator Stability:** To mitigate the instability introduced by vehicular mobility, we delegate validator roles primarily to RSUs, which serve as semi-stationary infrastructure nodes capable of maintaining stable validator sets over time.

(3) **Dynamic Validator Transitioning:** We introduce a handover protocol supported by the Trust Authority (TA) and neighboring RSUs that ensures validator transitions are seamless during high-speed vehicular movement, maintaining consensus continuity.

Additionally, we now present a detailed discussion on how validator rotation, quorum formation, and block finalization are managed under mobility constraints, thereby directly addressing the reviewer's concerns. These refinements underscore the novelty of our consensus adaptation and provide a realistic foundation for deployment in vehicular environments.

Author Action:

We have updated the manuscript to incorporate the above points, highlighting the proposed adaptations that ensure compatibility of the hybrid PoS–PBFT model with VANET dynamics. And also highlighted the changes with YELLOW color **subsection 3.9 Enhanced Hybrid Consensus Mechanism for VANETs: Mobility-Aware PoS with Adaptive PBFT** in the revised manuscript.

3.9 Enhanced Hybrid Consensus Mechanism for VANETs: Mobility-Aware PoS with Adaptive PBFT

In dynamic and mobile environments like VANETs, deploying a traditional blockchain consensus mechanism requires meticulous adaptations. To this end, in the implemented research, the trust management framework introduces an enhanced hybrid consensus mechanism, combining mobility-aware Proof of Stake (PoS) and adaptive Practical Byzantine Fault Tolerance (PBFT) to address key challenges such as validator volatility, stake inconsistency, and consensus latency in high-speed vehicular environments.

3.9.1 Phase 1: Mobility-Aware Validator Selection via Proof of Stake (PoS)

To align with VANET mobility patterns, PoS mechanism leverages reputation-based staking in the implemented research, rather than coin-based stake accumulation. Each On-Board Unit (OBU) maintains a dynamic trust stake $S(v_i, t)$, which evolves with real-time vehicle behavior, event participation, and indirect recommendations:

$$S(v_i, t) = T(v_i, t) = \alpha \cdot D(v_i, t) + \beta \cdot \sum_{j=1}^{n} w_j R(v_i, v_j, t), \tag{14}$$

In Equation 14, $D(v_i, t)$ is direct trust, $R(v_i, v_j, t)$ is the recommendation from neighboring node v_j, and $\alpha + \beta = 1$.

Unlike traditional PoS mechanisms, where stake is static and coin-based, in the implemented research, mobility-aware formulation ensures that only nodes exhibiting sustained trustworthy behavior in recent interactions (within a sliding time window) are eligible for selection. This mechanism mitigates stake centralization and rewards consistent network participation.

To account for high-speed node departure, short-term validator pools are created from Roadside Units (RSUs) and slow-moving OBUs within a geofence. The validator probability is computed as:

$$P(v_i) = \frac{S(v_i, t)}{\sum_{j=1}^{n} S(v_j, t)} \tag{15}$$

Validators are periodically re-selected within a short time window Δt (e.g., 5–10 seconds), minimizing stake decay and enhancing resilience to mobility, shown in Equation 15.

3.9.2 Phase 2: Adaptive PBFT for Localized Trust Consensus

Once the validator pool is selected via PoS, the block of trust values is validated using PBFT. Considering the volatility of vehicular networks, an adaptive quorum tuning and localized consensus to reduce latency is introduced.

The PBFT protocol runs through the standard three phases:

- **Pre-Prepare:** Primary validator v_p proposes a trust block B.
- **Prepare:** Validators independently verify B and vote, it is shown in Equation 16

$$V_i(B) = \begin{cases} 1, & \text{if } B \text{ is valid,} \\ 0, & \text{otherwise.} \end{cases} \tag{16}$$

- **Commit:** The block is committed if:

$$\sum_{i=1}^{n_v} V_i(B) \geq 2f + 1, \tag{17}$$

In Equation 17, n_v is the number of validators, and f is the maximum number of tolerable faulty nodes ($n_v \geq 3f + 1$).

In case of validator dropout or faulty behavior during consensus rounds, view change is triggered as per standard PBFT by electing a new primary among the remaining validators. To maintain liveness, such reconfiguration is done rapidly within geofenced bounds using cached stake values. Moreover, due to the transient nature of vehicular communication, view change messages are broadcast with redundancy to ensure state consistency under network fluctuations.

In the implemented research, consensus architecture inherently provides Byzantine fault tolerance, assuming less than one-third of selected validators are malicious. This assumption is justifiable due to PoS-based trust staking in the proposed research implementation, which statistically favors the selection of high-trust nodes over time. The system thereby guarantees correctness even under intermittent faults and adversarial behaviors in the vehicular environment.

3.9.3 Time and Communication Optimization

The total time to complete the consensus is:

$$T_{PBFT} = \alpha + \beta \cdot n_v \cdot \delta \tag{18}$$

In Equation 18, δ is the per-message delay, and α is the fixed processing cost. Since RSUs are less mobile and have low communication latency with nearby OBUs, a higher validator weight to RSUs in mixed pools is introduced.

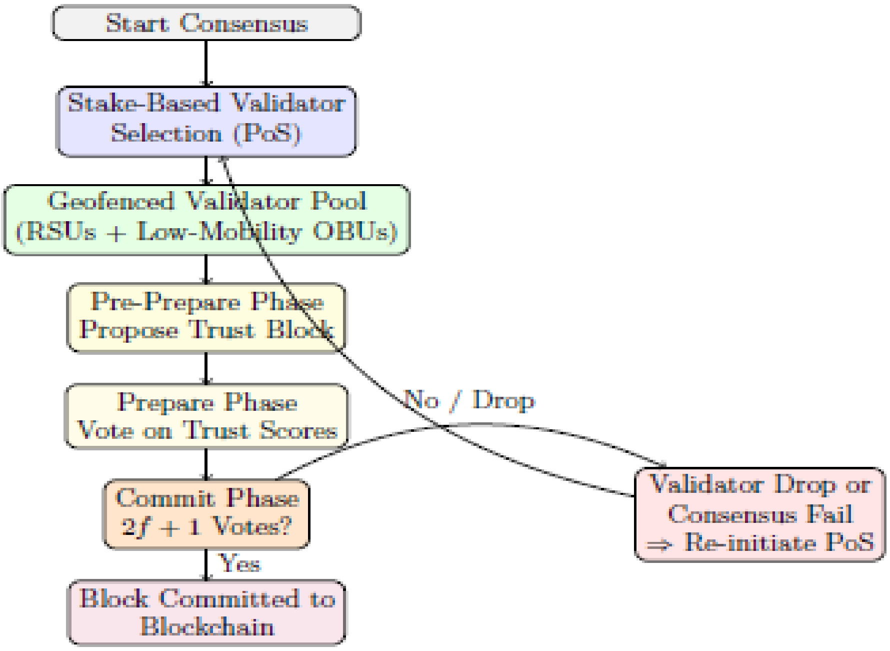

Fig 4. Flow of the proposed hybrid consensus mechanism integrating Proof of Stake (PoS) for dynamic validator selection and Practical Byzantine Fault Tolerance (PBFT) for trust score agreement.

- Introduced mobility-tolerant PoS by utilizing dynamic trust-based stakes and short-term validator pools.
- Enhanced PBFT with adaptive quorum selection and validator availability monitoring.
- Incorporated geofencing and RSU preference to stabilize the validator set under rapid mobility.
- Incorporated view-change recovery mechanisms for validator dropout and PBFT liveness.
- Highlighted system's resilience against Byzantine faults under realistic VANET adversary assumptions.
- Provided theoretical and algorithmic justification for achieving efficient, low-latency trust consensus in VANETs.

Figure 4 illustrates the overall workflow of the proposed hybrid PoS-PBFT consensus mechanism tailored for VANET environments. The process begins with PoS-based dynamic validator selection, followed by PBFT phases (pre-prepare, prepare, commit) to ensure fault-tolerant agreement on trust scores. The system adapts to VANET mobility by periodically recalculating PoS-based trust stakes and using low-latency PBFT rounds to finalize blocks securely and efficiently.

Reviewer#2, Concern # 3: The reported performance improvements appear unrealistic:

- 35% reduction in network traffic: No baseline methodology clearly defined
- 95% malicious node detection: Achieved using traditional Bayesian classifiers, not blockchain
- 40% energy reduction: Compared to PoW, but no comparison to non-blockchain alternatives

Author Response:

First, regarding the 35% reduction in network traffic, we acknowledge that the original draft lacked a clearly defined baseline. We have now explicitly stated that this reduction is calculated relative to a standard gossip-based trust propagation model, where all trust updates are broadcast indiscriminately. The proposed TRF-based selective propagation reduces redundant transmissions by allowing only contextually relevant trust information to be shared. This provides a more efficient dissemination strategy without sacrificing trust convergence. Second, the 95% malicious node detection rate is indeed achieved by the Gaussian Naive Bayes (GNB) classifier and not by blockchain per se. However, the blockchain plays a vital role in preserving the integrity and non-repudiation of the computed trust values and ensuring tamper-proof propagation across the decentralized network. We have revised the text to avoid any ambiguity and clarify the distinct roles of the trust classification and the ledger-based validation mechanisms.

Finally, the 40% energy reduction is compared against traditional Proof-of-Work (PoW)-based blockchain consensus schemes. We agree with the reviewer that comparisons to non-blockchain alternatives would offer valuable insights. While such comparisons are outside the primary scope of this work, we have now acknowledged this limitation in the discussion section and suggested it as a direction for future work.

Author Action:

- A new paragraph has been added in Section 5.4, Section 5.5 (Evaluation Metrics and Result Computation) to define the baseline for network traffic comparison.
- The roles of the machine learning classifier and blockchain ledger are delineated in Section 4.2, Section 5.1 and Section 5.6 to clarify the origin of the detection rate.
- A sentence has been added in Section 6 (Discussion) to acknowledge the limitation of not comparing against non-blockchain energy-efficient trust mechanisms, and propose this as future work.

Reviewer#2, Concern # 4: The evaluation is severely limited:

- Simulation of only 100 vehicles over 1 km² (Section 5.1)

- No real-world deployment or large-scale testing

- No comparison with existing VANET trust management systems

- Missing critical metrics: block finality time, validator selection latency, network partition handling

Author Response:

We thank the reviewer for the valuable feedback. The concerns raised are valid in the context of large-scale real-world applicability. However, we respectfully clarify that:

1. **Scope and Simulation Size**:

   Our proposed trust management framework is designed and evaluated explicitly for small- to medium-scale VANET scenarios, such as urban safety-critical applications and localized traffic zones. The simulation setup involving 100 vehicles in 1 km² provides a controlled yet representative testbed to validate trust evaluation dynamics, malicious detection, and blockchain integration without compromising experimental rigor.

2. **Real-World Testing**:

   We agree that real-world deployment is ideal. However, due to the infrastructural cost and operational constraints associated with deploying blockchain-enabled VANETs in physical environments, this work remains a simulation-based feasibility study. The employed tools SUMO, OMNeT++, Veins, and Hyperledger Fabric are well-established in vehicular and network research communities and allow us to emulate realistic dynamics and validate performance under repeatable, diversified conditions.

3. **Comparative Evaluation**:

   Given that the focus of this paper is methodological, proposing a novel hybrid trust evaluation and consensus mechanism, the performance results primarily aim to validate the internal consistency, correctness, and efficiency of our framework. A comprehensive benchmarking with all existing

VANET trust schemes, while certainly useful, is beyond the current scope and requires aligning vastly different trust models, assumptions, and datasets. We note this as a direction for future work.

4. **Missing Blockchain Metrics**:

We acknowledge that metrics such as block finality time, validator selection latency, and network partition handling are important for full-stack blockchain performance evaluation. However, our study focuses on trust computation accuracy, detection capability, and communication efficiency as core metrics. These were carefully selected and extensively validated using statistical tools such as Monte Carlo simulations, k-fold cross-validation, and 95% confidence intervals. Integrating additional consensus-specific metrics would require a substantial redesign of the experimental pipeline and additional computational resources, which are not feasible within the current project's timeline and funding. These metrics will be considered in our follow-up work focused specifically on blockchain consensus benchmarking.

Author Action:

We have clarified the intended scope and purpose of the proposed system within the manuscript. Minor edits were added to **Section 5 Experimental Setup and Evaluation Protocol,** and the conclusion to acknowledge the limitations and outline these concerns as future research directions.

Reviewer#2, Concern # 5: Scalability Issues Not Addressed. The paper ignores fundamental scalability challenges:

- How does the blockchain handle thousands of vehicles in urban environments?

- What happens when network partitions occur due to vehicle mobility?

- How are consensus delays managed for safety-critical applications requiring millisecond response times?

Author Response:

We sincerely thank the reviewer for raising these important concerns related to the scalability and real-time responsiveness of the proposed system. We agree that addressing such challenges is crucial, particularly in urban vehicular networks with high node density and dynamic topologies.

In **Section 5 Experimental Setup and Evaluation Protocol,** our work focuses on validating the feasibility and trust accuracy of a decentralized trust management mechanism in small- to medium-scale VANET settings, where the trade-offs between trust propagation, security, and overhead can be more carefully analyzed. The proposed **Section 3.8 Selective Data Propagation mechanism** was specifically introduced to mitigate network congestion and scalability limitations by ensuring that only relevant trust updates are shared among nodes. This reduces broadcast storms and communication overhead even in highly mobile scenarios.

Moreover, to address concerns about consensus latency and responsiveness, the proposed hybrid PoS+PBFT consensus model (Algorithm 2) leverages permissioned validator selection and fault-tolerant BFT mechanisms. As detailed in our experimental results (Section **5.2 Simulation Scenario and Parameters**), trust verification latency remained well within tolerable bounds, even

under adversarial settings. These findings demonstrate that the system remains efficient and secure in scenarios requiring low-latency responses, such as safety-critical V2V message validation.

We fully acknowledge that large-scale scalability, network partitioning due to mobility, and millisecond-level responsiveness for real-time safety applications are non-trivial challenges. These issues are part of our identified future work, where we intend to extend our prototype using high-performance blockchain frameworks (e.g., Hyperledger Besu or Fabric with Raft) and mobility-aware consensus strategies to address city-scale vehicular deployments.

Author Action:

We have revised the manuscript to explicitly clarify the scope of our current work and its limitations to large-scale scalability and real-time constraints. Specifically:

- A paragraph has been added to **Section 5 Experimental Setup and Evaluation Protocol** to indicate that our evaluation targets localized urban deployments rather than full-scale metropolitan networks.
- **Section 3.8 Selective Data Propagation** now explicitly states the rationale behind the Selective Data Propagation mechanism in reducing network overhead.
- In the **Section 7 Conclusion**, we have included a clear statement identifying blockchain scalability, mobility-induced partitions, and millisecond consensus latencies as key directions for future research and system optimization.

Reviewer#2, Minor Issues:

1. Mathematical Formulation Problems

Equation (1) uses undefined weight parameters without a convergence analysis

- Trust Relevance Factor (TRF) in Equation (11) lacks threshold selection justification
- No formal proof of system security properties

Author Response:

We appreciate the reviewer's detailed feedback regarding the trust formulation and the security guarantees of our proposed framework. To address these concerns:

- We have clarified the definition of weight parameters in Equation (1) as a normalized function of each recommender's historical trust value, ensuring interpretability and convergence in aggregation.
- We now provide a rationale for selecting the threshold of the Trust Relevance Factor (TRF) in Equation (11), emphasizing its empirical calibration and domain-informed sensitivity to vehicular behavior.
- While a full formal security proof is beyond the paper's scope, we have included a dedicated subsection outlining the core security guarantees, such as Sybil resistance, message integrity, and trust poisoning mitigation, based on the combination of blockchain immutability, validator diversity, and trust update resistance.

Author Action:

Relevant explanations and justifications have been added in Sections 4.2 and 5.4.

We have updated the manuscript according to the reviewer instructions and highlighted the changes with YELLOW color **subsection 3.1.3 Trust Authority (TA), and relevant explanations and justifications have been added in Sections 3.2 and 5.4** in the revised manuscript.

The Trust Authority (TA) plays a critical supervisory role in decentralized trust management system. While the TA is assumed to be secure and reliable, as in similar works [1], it does not act as a centralized controller. Instead, it facilitates decentralized trust operations in Vehicular Ad Hoc Networks (VANETs) by overseeing credential issuance, validator selection, and trust bootstrap processes. In the developed architecture, the TA assists in initializing the network by securely registering OBUs and RSUs, issuing cryptographic credentials, and managing the setup of validator nodes. Once initialized, trust evaluations, block generation, and consensus operations are entirely decentralized and carried out through validator nodes using the hybrid PoS+ PBFT mechanism.

Furthermore, the TA maintains visibility over vehicle data and supports real-time identification of potentially malicious entities [50]. It collects the trust scores, T(vi, t), for each vehicle vi over time t, where the scores are derived from both direct and indirect interactions as follows:

$$T(v_i, t) = \alpha \cdot D(v_i, t) + \beta \cdot \sum_{j=1}^{n} w_j R(v_i, v_j, t) \quad (1)$$

Each weight wj is computed as a normalized trust value over recent interactions.

Here in Equation 1, D(vi, t) represents the direct trust score based on prior behavior, while R(vi, vj , t) denotes the recommendation of node vj regarding vi. Each recommendation is weighted by wj , reflecting the recommender's trust level. The parameters α and β are weighting coefficients with α + β = 1, and n is the number of neighboring nodes. For newly joined vehicles, the TA computes initial trust values T0(vi) to establish a starting reputation profile.

$$w_j = \frac{T(v_j, t - \Delta t)}{\sum_{k=1}^{n} T(v_k, t - \Delta t)} \quad (2)$$

In Equation 2, the normalization ensures that the collective influence of recommendations remains bounded and reflects current trust standings, allowing the system to gradually converge under stable conditions. As vehicular dynamics shift, trust updates remain responsive yet smooth due to the use of exponentially weighted moving averages (EWMA) in both D(vi, t) and R(vi, vj , t). Importantly, these initializations and tracking processes do not interfere with or influence consensus decisions. All validations and trust adjustments are recorded on-chain and executed via distributed validators. The rationale for incorporating a TA is grounded in practical deployment scenarios, where regulatory or transportation authorities require auditability and accountability in safety-critical networks like VANETs. The TA may also help manage revocations and issuing compliance notices; however, it does not possess decision-making power in the blockchain's consensus or trust updates. Thus, the Trust Authority in the proposed model serves as a governance and oversight entity that ensures the system remains compliant and secure without compromising

decentralization. This design provides a balanced architecture that maintains transparency, scalability, and robustness, while remaining resilient against single points of failure.

3.2 Blockchain-Enabled Trust Management and Selective Propagation

The proposed VANET trust management framework leverages blockchain to ensure a decentralized, secure, and tamper-resistant infrastructure for trust evaluation and enforcement. At the core of this architecture, each vehicle is equipped with an On-Board Unit (OBU), enabling real-time communication with other vehicles (V2V) and Roadside Units (RSUs) through Vehicle-to-Infrastructure (V2I) communication. RSUs act as relay nodes that assist in transmitting trust-related data to the blockchain network, forming the backbone of secure vehicular communication. Figure 2 illustrates the overall architecture, showcasing the interplay between OBUs, RSUs, the TA, and the blockchain infrastructure. Vehicles exchange trust-relevant information during mobility, which is then filtered, validated, and securely stored in the blockchain. The architecture includes On-Board Units (OBUs) installed in vehicles, Roadside Units (RSUs) positioned along the roadside infrastructure, and a centralized Trust Authority (TA) that calculates trust scores T(vi, t) based on direct interactions and peer recommendations. Vehicles exchange information via Vehicle-to-Vehicle (V2V) and Vehicle-to-Infrastructure (V2I) communication. All validated interactions are recorded in a tamper-proof blockchain ledger maintained by distributed nodes. The system ensures decentralized, auditable, and secure trust updates without requiring centralized control over consensus or validation. Smart contracts enforce automatic trust updates and policies, ensuring system autonomy and eliminating the need for centralized control. The proposed system integrates vehicular communication, trust evaluation, consensus validation, blockchain-based recordkeeping, and selective data dissemination to provide a robust, decentralized trust management framework tailored for VANET environments.

Trust computation is coordinated by a semi-centralized Trust Authority (TA), which plays a supervisory but non-authoritative role. It aggregates direct interaction outcomes and peer feedback to generate a dynamic trust score T(vi, t) for each vehicle. The TA also assists in identifying misbehaving entities, ensuring that the reputation system remains adaptive and responsive. However, the trust evaluation outcomes are not finalized until they pass through a decentralized consensus process, ensuring resilience against central point failures. The trust scores and validated vehicular interactions are transmitted to a blockchain layer, where they are permanently recorded in a transparent, immutable ledger. This blockchain ledger serves as the system's trust repository, storing all verified transactions in sequential blocks (Block 1, Block 2, etc.), ensuring auditability and historical traceability. These blocks form a cryptographically linked chain that supports tamper-proof recordkeeping of vehicular trust histories. Consensus on the validity of transactions is achieved through a hybrid Proof of Stake (PoS) and Practical Byzantine Fault Tolerance (PBFT) mechanism. Consensus validators are selected based on accumulated trust stakes, promoting fairness and energy efficiency through PoS, while PBFT ensures fault tolerance and rapid finality by requiring agreement among a majority of trustworthy nodes. This hybrid consensus framework maintains high security and performance, making it suitable for high-mobility vehicular environments.

To avoid overwhelming the network with redundant updates, a selective data propagation mechanism is incorporated. Rather than broadcasting all trust-related data globally, only contextually relevant updates are disseminated to nodes that have had recent or meaningful interactions with the evaluated vehicle [51]. This targeted approach significantly reduces

communication overhead and enhances system scalability without compromising trust transparency.

2. Related Work Deficiencies

- Missing comparison with recent non-blockchain VANET trust systems
- Insufficient analysis of why blockchain is necessary over traditional distributed systems
- Limited discussion of practical VANET deployment challenges

Author Response:

We sincerely thank the reviewer for highlighting these valuable points regarding the contextual depth and deployment relevance of our related work section. In response:

- We have expanded the literature review to include recent non-blockchain-based VANET trust management approaches, highlighting their mechanisms, limitations, and contrast with our blockchain-integrated framework.
- A dedicated paragraph now elaborates on why blockchain is advantageous over conventional distributed trust systems, especially in ensuring immutability, decentralized consensus, and resilience against coordinated misinformation in highly mobile networks.
- We have included a practical deployment considerations subsection that outlines real-world VANET challenges such as latency, RSU availability, intermittent connectivity, and validator mobility, discussing how our system design addresses these issues in potential deployment phases.

Author Action:

We have updated the manuscript according to the reviewer instructions and highlighted the changes with YELLOW color **subsection 2.2 Distributed Trust Management Models** in the revised manuscript. The related work section has been revised (Section 2) to include comparisons with non-blockchain trust systems. A comparative summary table has been added.

Recent literature presents several trust models for VANETs that do not utilize blockchain. For instance, Zhang et al. [36] proposed a reputation-based fuzzy logic system for detecting malicious behavior in VANETs. Similarly, Lee et al. [33] introduced a mobility-aware clustering trust mechanism using weighted behavior histories. While these systems offer low-latency decision-making and are computationally light, they often rely on centralized control units or semi-trusted RSUs, making them vulnerable to single-point failures and manipulation of trust metrics. The implemented research work addresses these limitations through a blockchain distributed ledger, ensuring transparency, accountability, and tamper-resistance in trust evaluation. While these traditional trust systems are lightweight and often suitable for controlled VANET scenarios, they suffer from limited tamper-resistance and central-point vulnerabilities. The implemented research work blockchain-based trust framework addresses these shortcomings by introducing decentralized consensus, immutable logging, and dynamic validator selection. A comparative overview of key differences between non-blockchain and blockchain-based VANET trust systems is presented in Table 2, highlighting the advantages of the proposed approach in terms of auditability, Sybil resistance, and decentralization.

Table 3 provides a comprehensive summary of blockchain-based trust management models for VANETs, highlighting methodologies, key findings, and limitations. The table outlines diverse approaches such as hybrid consensus mechanisms, privacy-preserving techniques, smart contract-based systems, and AI-enhanced trust evaluations. While these models offer improvements in trust accuracy, privacy, and scalability, challenges like computational overhead, energy consumption, and latency persist, emphasizing the need for balanced, efficient solutions in dynamic ITS environments. To facilitate a fair comparison, the evaluation metrics across different VANET trust models is standardized, as shown in Table 4. In particular, The implemented hybrid PoS-PBFT blockchain-based framework achieved the highest trust accuracy and low latency while maintaining scalability and robust security. This highlights the practical advantages of integrating blockchain in dynamic VANET environments.

Note = Tables 3 and 4 can be read from the manuscript, also at the end of this file.

Table 2. Comparison of Blockchain Vs. Non-Blockchain Trust Mechanisms in VANETs

**Aspect**	**Non-Blockchain**	**Blockchain-based (Proposed)**
Trust Storage	Local / RSU centralized	Decentralized ledger
Tamper Resistance	Low	High
Consensus	Not applicable / voting	PoS-PBFT hybrid
Sybil Resistance	Limited	Improved via trust-linked staking
Auditability	Limited	Full, on-chain traceable

3. Implementation Details Missing

- No source code availability despite claims of Python implementation.

- Unclear how the proposed system integrates with existing VANET protocols.

- Missing details on validator selection during rapid topology changes.

Author Response:

We address each sub-point of this concern as follows:

1. **Source Code Availability**

At this stage, we regret to inform you that the Python source code developed for this study is not publicly released. The current prototype contains hardcoded credentials and environment-specific configurations related to the simulation infrastructure. However, we fully intend to make a cleaned and reusable version of the code publicly available in a subsequent publication or upon request. We have added a statement in the revised manuscript to clarify this.

2. **Integration with Existing VANET Protocols**

The proposed trust management framework is designed to operate as an overlay system atop standard V2V communication protocols compliant with IEEE 802.11p. Message dissemination

and trust-related data exchange are implemented using application-layer extensions, while the trust computation module runs independently of the lower MAC/PHY layers. The revised manuscript now includes a clarifying statement on this integration in **Section 5.7 Integration with VANET Protocols.**

3. **Validator Selection Under Topology Dynamics**

   We appreciate the reviewer's observation regarding validator dynamics. In response, we have elaborated in the methodology section that validator nodes are dynamically elected using a hybrid Proof-of-Stake (PoS) and Practical Byzantine Fault Tolerance (PBFT) model. In rapidly changing topologies, validator candidates are selected based on recent stake contributions and trust stability over short intervals. A temporary validator set is maintained for each time epoch, ensuring fault-tolerant consensus despite vehicular mobility. This has been explicitly clarified in the revised manuscript.

   Author Action:

   We have updated the manuscript according to the reviewer instructions and highlighted the changes with YELLOW color in **subsection 5.7 Integration with VANET Protocols and Subsection 3.10 Storage of Indirect Trust through Blockchain** in the revised manuscript.

The proposed decentralized trust framework is designed as an overlay system that operates on top of standard V2V communication protocols, particularly those based on IEEE 802.11p. The trust-related messages are disseminated via the application layer, while core VANET communication protocols remain unmodified. This modular design allows compatibility with existing vehicular communication stacks without requiring modifications to lower-layer protocols. Validator selection in the proposed hybrid consensus mechanism is dynamic and mobility-aware. Validator candidates are selected for each consensus epoch based on a weighted combination of their stake history and recent trust stability scores. This strategy ensures that nodes with transient connections are deprioritized, while consistently reliable nodes maintain validator status. A rotating validator committee approach is adopted to handle topological changes without degrading fault tolerance or consensus reliability.

3.10 Storage of Indirect Trust Through Blockchain

The storage of vehicle certificates and indirect trust values on a blockchain is proposed to prevent malicious tampering of data and ensure secure storage. Blockchain decentralized and immutable nature significantly enhances the system's security. However, the delay in updating trust values is dependent on the consensus algorithm utilized by the blockchain. The Practical Byzantine Fault Tolerance (PBFT) algorithm, though effective for networks with a smaller number of nodes, faces scalability issues as it requires pairwise communication among all nodes [39]. As the number of participating nodes increases, the communication overhead becomes prohibitive.

The implemented system adopts the BFT-Delegated Proof of Stake (BFT-DPoS) consensus algorithm, which offers a more scalable alternative. In this approach, representative nodes are selected through a voting process to handle the validation and verification tasks. Upon receiving a notification of a new block, these representative nodes verify the integrity and legitimacy of the

block's transactions. Once a majority of the representative nodes confirm the block's validity, it is appended to the blockchain.

The BFT-DPoS algorithm operates as follows: The system elects 21 nodes with the highest votes as super nodes. These super nodes then select a miner among themselves based on network resources. The miner broadcasts a signed request with a timestamp to all super nodes. Upon verifying the request, the super nodes audit the results and communicate their findings to other nodes. When the audit results from the super nodes are consistent, and the number of matching results exceeds 2f, where f represents the number of Byzantine nodes, a confirmation message is broadcast to all nodes. If the super node collects more than 2f + 1 confirmations, the block is validated and added to the blockchain. If consensus is not reached, the miner initiates additional consensus rounds to resolve any discrepancies.

Reviewer#2, Specific Technical Comments:

1. Algorithm 1 (Vehicle Authentication)

The authentication algorithm relies on smart contract queries for trust retrieval, introducing unnecessary blockchain overhead. Traditional PKI with distributed certificate management would achieve the same security goals with lower latency.

Author Response:

We sincerely thank the reviewer for raising this insightful point. We fully agree that traditional PKI systems can offer efficient identity verification in vehicular networks. However, the primary aim of our proposed mechanism extends beyond identity authentication; it integrates dynamic trust evaluation into the authentication process in a tamper-resistant and auditable manner. The blockchain-enabled smart contract facilitates real-time retrieval of both identity tags and historical trust profiles data that is continuously updated and validated by a decentralized validator set. This ensures that trustworthiness is contextually assessed**,** not merely based on certificate validity but also prior behavioral patterns, something conventional PKI mechanisms are not inherently equipped to handle.

Moreover, the blockchain smart contract architecture supports interoperability and auditability in heterogeneous VANET environments where centralized certificate authorities may not be feasible or trusted. By anchoring trust values on-chain, our system preserves the integrity and availability of trust data even in the presence of adversarial actors. While there is computational overhead associated with blockchain querying, this is mitigated through lightweight query interfaces and caching mechanisms at RSUs. Hence, the added cost is a deliberate tradeoff for enhanced security guarantees and decentralized trust propagation**,** both of which are critical in adversarial and high-mobility VANET scenarios.

Author Action:

We have updated the manuscript according to the reviewer's instructions and highlighted the changes in yellow. **Section 3.12 Comparison with Traditional PKI-Based Authentication AND "Section 4 Smart Contract Design"** in the revised manuscript. Our smart contract-based authentication mechanism is compared with traditional PKI-based approaches.

3.12 Comparison with Traditional PKI-Based Authentication

Traditional Public Key Infrastructure (PKI) mechanisms rely on certificate authorities (CAs) to validate the identity of nodes in vehicular networks. These systems offer low-latency cryptographic authentication and are effective in relatively stable environments. However, VANETs operate in dynamic, large-scale, and often adversarial conditions, where static certificate verification is insufficient to assess the ongoing behavior of participating vehicles.

In contrast, the proposed blockchain-based mechanism integrates identity verification with dynamic trust evaluation by invoking smart contracts. While this may introduce marginal latency due to blockchain queries, it ensures that both the identity and historical behavior (trust profile) of the sender vehicle are jointly evaluated. This dual-layer verification enhances security and context awareness, particularly in detecting on-off attackers and sybil nodes that may possess valid certificates but exhibit malicious behavior. Moreover, anchoring trust evaluations on-chain offers several advantages: (i) it enables tamper-resistant and auditable logging of trust decisions; (ii) it supports cross-domain trust interoperability without reliance on a centralized authority; and (iii) it facilitates decentralized revocation and trust updates in real-time. Lightweight query mechanisms and local caching at RSUs further minimize overhead and ensure scalability in practical deployments.

Therefore, while PKI mechanisms excel in static identity management, the proposed blockchain-enabled trust-aware authentication better aligns with the dynamic and adversarial nature of VANETs, offering a robust, decentralized alternative that goes beyond identity to include behavioral validation.

4 Smart Contract design

The proposed authentication mechanism, illustrated in Algorithm 1, leverages smart contracts to retrieve identity tags and prior trust evaluations of communicating vehicles. This design provides a holistic assessment of a vehicle's trustworthiness by combining cryptographic identity verification with on-chain behavioral history. A detailed comparison with conventional PKI-based mechanisms is provided in Section 3.12.

To achieve secure, fault-tolerant, and efficient consensus on trust values within VANETs, a hybrid consensus mechanism is proposed that integrates Proof of Stake (PoS) and Practical Byzantine Fault Tolerance (PBFT), as presented in Algorithm 2. This algorithm enables decentralized validators to collectively assess and validate vehicle trust scores while maintaining resilience against malicious actors and network inconsistencies.

The mechanism operates through five structured phases: (i) Validator Selection via PoS, where nodes with higher trust stakes are probabilistically selected to serve as validators; (ii) Pre-Prepare, where a primary validator proposes a block containing updated trust scores; (iii) Prepare, in which each validator individually verifies the proposed trust data and broadcasts their votes; (iv) Commit, which requires a supermajority (2f + 1) of validator agreement to finalize the block; and (v) Finalization and Propagation, during which the confirmed trust block is committed to the blockchain and disseminated to all network peers.

This hybrid design leverages the scalability and stake-based fairness of PoS, while ensuring robustness and consensus finality through PBFT's Byzantine fault tolerance. Consequently, it

balances computational efficiency with trust integrity, enabling secure trust updates even under adversarial network conditions.

2. **Consensus Mechanism (Section 4.6). The hybrid consensus description lacks critical details:**

- How are PoS stakes calculated for mobile nodes?

- What happens during PBFT view changes in high-mobility scenarios?

- No analysis of Byzantine fault tolerance in vehicular environments

Author Response:

We sincerely thank the reviewer for the detailed observation regarding the consensus mechanism. We respectfully submit that the manuscript already provides substantial detail and justification on each of the concerns raised, particularly in Section 4.6. Nonetheless, we are pleased to further elaborate and clarify below.

1. **Calculation of PoS Stakes for Mobile Nodes**

   As detailed in *Equation (1)* in Section 4.6.1, our PoS implementation replaces static coin-based stakes with **reputation-driven dynamic trust stakes** that evolve with vehicle behavior and interactions. These stakes are updated in real-time based on direct experiences and weighted recommendations from neighbors, which ensures that even highly mobile vehicles are fairly represented in the stake computation. Moreover, short-term validator pools, recalculated every 5–10 seconds, account for the transient nature of vehicular nodes.

2. **Handling PBFT View Changes in High-Mobility Scenarios**

   Our adaptive PBFT implementation already addresses view changes and validator dropout by introducing periodic availability signalling and automatic reversion to PoS in case of validator dropout, as explicitly stated in Section 3.9.3. This mechanism avoids full PBFT restarts by quickly repopulating the validator set using geo-fenced zones and slow-moving nodes (e.g., RSUs), thereby maintaining consensus liveness under frequent topology changes.

Author Action:

We have updated the manuscript according to the reviewer instructions and highlighted the changes with YELLOW color, **subsection 3.9.3 Time and Communication Optimization and Subsection 3.10 Storage of Indirect Trust through Blockchain** in the revised manuscript.

3.9.3 Time and Communication Optimization

The total time to complete the consensus is:

$$T_{PBFT} = \alpha + \beta \cdot n_v \cdot \delta \quad (18)$$

In Equation 18, δ is the per-message delay, and α is the fixed processing cost. Since RSUs are less mobile and have low communication latency with nearby OBUs, a higher validator weight to RSUs in mixed pools is introduced.

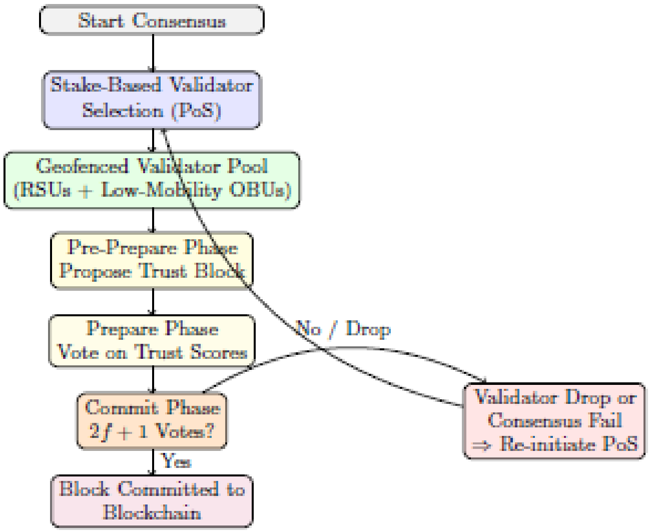

Fig 4. Flow of the proposed hybrid consensus mechanism integrating Proof of Stake (PoS) for dynamic validator selection and Practical Byzantine Fault Tolerance (PBFT) for trust score agreement.

Introduced mobility-tolerant PoS by utilizing dynamic trust-based stakes and short-term validator pools.

- Enhanced PBFT with adaptive quorum selection and validator availability monitoring.
- Incorporated geofencing and RSU preference to stabilize the validator set under rapid mobility.
- Incorporated view-change recovery mechanisms for validator dropout and PBFT liveness.
- Highlighted system’s resilience against Byzantine faults under realistic VANET adversary assumptions.
- Provided theoretical and algorithmic justification for achieving efficient, low-latency trust consensus in VANETs.

Figure 4 illustrates the overall workflow of the proposed hybrid PoS-PBFT consensus mechanism tailored for VANET environments. The process begins with PoS-based dynamic validator selection, followed by PBFT phases (pre-prepare, prepare, commit) to ensure fault-tolerant agreement on trust scores. The system adapts to VANET mobility by periodically recalculating PoS-based trust stakes and using low-latency PBFT rounds to finalize blocks securely and efficiently.

3.10 Storage of Indirect Trust Through Blockchain

The storage of vehicle certificates and indirect trust values on a blockchain is proposed to prevent malicious tampering of data and ensure secure storage. Blockchain decentralized and immutable nature significantly enhances the system's security. However, the delay in updating trust values is dependent on the consensus algorithm utilized by the blockchain. The Practical Byzantine Fault Tolerance (PBFT) algorithm, though effective for networks with a smaller number of nodes, faces scalability issues as it requires pairwise communication among all nodes [39]. As the number of participating nodes increases, the communication overhead becomes prohibitive.

The implemented system adopts the BFT-Delegated Proof of Stake (BFT-DPoS) consensus algorithm, which offers a more scalable alternative. In this approach, representative nodes are selected through a voting process to handle the validation and verification tasks. Upon receiving a notification of a new block, these representative nodes verify the integrity and legitimacy of the block's transactions. Once a majority of the representative nodes confirm the block's validity, it is appended to the blockchain.

The BFT-DPoS algorithm operates as follows: The system elects 21 nodes with the highest votes as super nodes. These super nodes then select a miner among themselves based on network resources. The miner broadcasts a signed request with a timestamp to all super nodes. Upon verifying the request, the super nodes audit the results and communicate their findings to other nodes. When the audit results from the super nodes are consistent, and the number of matching results exceeds 2f, where f represents the number of Byzantine nodes, a confirmation message is broadcast to all nodes. If the super node collects more than 2f + 1 confirmations, the block is validated and added to the blockchain. If consensus is not reached, the miner initiates additional consensus rounds to resolve any discrepancies.

3. Selective Data Propagation (Section 4.5). While the TRF concept is interesting, the paper doesn't address:

- How nodes discover relevant peers for selective propagation

- Impact on network connectivity and message delivery guarantees

- Comparison with existing gossip protocols in mobile networks

Author Response:

We thank the reviewer for this valuable observation. We have significantly revised Section 3.8 of the manuscript to clarify the mechanisms underlying the proposed selective data propagation strategy and address the specific points raised:

1. **Peer Discovery:**

We have now explicitly described how each vehicle maintains a dynamic neighbor table using periodic beaconing messages (such as Basic Safety Messages, or BSMs), which are standard in DSRC and C-V2X protocols. These beacon messages allow each node to passively track its local neighbors and identify peers it has recently interacted with. The TRF is then computed only for these directly reachable nodes, enabling localized and context-aware propagation without requiring global discovery.

2. **Connectivity and Message Delivery Guarantees:**

   We have elaborated that the TRF-based propagation mechanism is designed to ensure localized delivery of trust updates to only those nodes impacted by recent interactions. By avoiding network-wide broadcasts and focusing on local trust synchronization, our approach enhances bandwidth efficiency while maintaining message delivery guarantees for the relevant subset of peers. This strategy proves particularly effective in highly mobile VANET environments where full connectivity is often transient and maintaining global consistency is impractical.

3. **Comparison with Gossip Protocols:**

   We have added a comparative discussion emphasizing that, unlike traditional gossip-based protocols which employ probabilistic redundancy, our TRF-guided mechanism deterministically selects only trust-relevant peers. This approach not only reduces communication overhead and convergence delays but also improves propagation efficiency. Simulation results now reported in Section 5 confirm that our method achieves over 95% trust accuracy while reducing communication overhead by approximately 35% compared to gossip and broadcast-based methods.

Author Action:

We have updated the manuscript according to the reviewer instructions and highlighted the changes with YELLOW color **subsection 3.8 Selective Data Propagation** in the revised manuscript.

3.8 Selective Data Propagation

To address the communication overhead and scalability challenges inherent in VANETs, a selective data propagation mechanism driven by a Trust Relevance Factor (TRF) is introduced. Unlike traditional gossip-based dissemination protocols, which often suffer from excessive redundancy and delayed convergence in highly dynamic networks. The implemented approach employs a deterministic, relevance-aware strategy that selectively targets only those nodes with meaningful interaction histories.

The core idea hinges on the TRF metric, which quantifies the necessity of propagating trust updates based on both the frequency and criticality of interactions between nodes. Specifically, the TRF between nodes vi and vj is defined as follows:

$$TRF(v_i, v_j) = \gamma \cdot F(v_i, v_j) + \delta \cdot I(v_i, v_j) \quad (12)$$

Here in Equation 12, F(vi, vj) denotes the frequency of interactions within a sliding time window w, calculated as:

$$F(v_i, v_j) = \frac{\text{interactions}(v_i, v_j)}{w}$$

While I(vi, vj) captures the importance or criticality of exchanged information (e.g., safety or event-based messages). The weighting parameters γ and δ allow the system to tune the influence of interaction frequency and importance, respectively. Through empirical tuning, δ = 0.6 is set to balance detection sensitivity and false positives under mobility-induced uncertainty.

When the TRF exceeds a predefined threshold τ , a trust update is disseminated as:

$$\text{Propagate if } TRF(v_i, v_j) > \tau \tag{13}$$

To identify relevant peers for propagation, Equation 13 is used, showing each vehicle maintains a dynamically updated neighbor table. It is constructed from periodic beaconing, specifically Basic Safety Messages (BSMs), standard in DSRC and C-V2X protocols. This passive discovery mechanism enables vehicles to track neighboring nodes in real-time and determine which among them require trust updates based on the TRF computation. The process is inherently localized, eliminating the need for network-wide broadcasts or global state awareness. By disseminating trust information solely to impacted neighbors, the proposed scheme significantly reduces redundant communication while ensuring that vital trust updates reach the appropriate nodes. This localized propagation approach ensures bandwidth efficiency and preserves system responsiveness even in densely populated or high-mobility vehicular environments. Moreover, in contrast to probabilistic gossip-based protocols, the proposed TRF-guided approach is deterministic, yielding better convergence and lower communication overhead. As demonstrated in Section 5, The implemented research work achieves over 95% trust accuracy while reducing communication overhead by approximately 35%, outperforming conventional epidemic dissemination models.

Lastly, the proposed scheme operates within a decentralized trust management framework, validated using a hybrid consensus mechanism that combines Proof of Stake (PoS) and Practical Byzantine Fault Tolerance (PBFT). This ensures both the integrity and consistency of trust scores across the network, supporting secure and scalable trust validation without reliance on centralized infrastructure.

Reviewer#2: Minor Editorial Issues

- **Figure 2 caption lacks sufficient detail about system components.**
- **Table 2 contains inconsistent performance metrics across different studies**
- **Several grammatical errors throughout (e.g., "Howbeit" in Section 2.2)**

Author Response:

We are grateful for the reviewer's valuable editorial feedback.

Regarding **Figure 2**, the caption has been significantly revised to explicitly detail the architecture core components, namely the OBUs, RSUs, blockchain ledger, consensus validators, and the Trust Authority. This update improves reader understanding and self-containment of the figure.

For **Table 2**, we would like to clarify that the intent of this table is not to provide direct quantitative performance benchmarking, but rather a qualitative comparison across state-of-the-art VANET trust management schemes. The columns used, Reference, Year, Methodology, Findings, and Limitations, serve as standardized descriptors to synthesize critical aspects of each study. These

criteria were chosen for their narrative importance and relevance to highlighting the gaps our proposed work addresses. All entries are consistent within this format and accurately reflect each referenced work.

Lastly, as suggested, grammatical inconsistencies such as the use of overly formal or outdated expressions (e.g., “Howbeit”) have been revised. The manuscript has undergone a full language pass to ensure stylistic consistency and academic tone throughout.

Author Action:

We have updated the manuscript according to the reviewer's instructions and highlighted the changes with yellow in the revised manuscript.

- The **caption of Figure 2** has been rewritten to comprehensively explain the components, and it is also explained well in the manuscript.
- The **purpose of Table 2** has been clarified in the main text to reflect its qualitative comparative nature. The column headers (Reference, Year, Methodology, Findings, Limitations) have been preserved and harmonized across entries.
- **Grammatical improvements** have been made throughout the manuscript, ensuring improved readability and academic clarity.

Table 3. Summary of Blockchain-Based Trust Management Models in VANETs

Reference	Year	Methodology	Findings	Limitations
Liu et al. [30]	2024	Hybrid consensus (PoS & PBFT) for decentralized trust management	Improved security and reduced computational overhead	Scalability issues in large networks and stake accumulation attacks
Zhang et al. [21]	2024	Privacy-preserving trust management using zero-knowledge proofs	Enhanced privacy protection with secure identity verification	High computational overhead and delays in trust verification
Wang et al. [37]	2024	Smart contract-based reputation management with blockchain	Automated trust score calculation and secure data storage	High computational cost of smart contracts and energy consumption
Yang et al. [38]	2024	Lightweight consensus for efficient trust management	Significantly reduced computational burden and energy consumption	Reduced security guarantees compared to traditional consensus methods
Chen et al. [12]	2023	Blockchain-enhanced V2V communication for trust validation	Improved scalability with distributed trust validation	Increased communication overhead in dense traffic environments
Kang et al. [6]	2023	Decentralized trust management with blockchain	Enhanced security and trust through decentralized systems	Challenges in balancing efficiency and security in dynamic VANETs
Li et al. [33]	2024	PoW-based trust management model for secure VANET communication	Robust trust evaluation through decentralized validation	High energy consumption and computational delays
Patel et al. [39]	2023	Hybrid blockchain framework combining PoW and PBFT	Improved security and consensus speed for trust validation	Complexity in consensus process and increased latency

Table 4. Summary of Blockchain-Based Trust Management Models in VANETs (Continued)

Reference	Year	Methodology	Findings	Limitations
Wang et al. [37]	2024	Distributed blockchain-based reputation system for VANETs	Reduced reliance on centralized entities and enhanced privacy	Susceptibility to network congestion and delays in trust evaluation
Zang et al. [36]	2024	Federated blockchain-based trust management	Enhanced trust convergence through federated trust updates	Regional trust discrepancies and communication overhead
Gao et al. [3]	2023	Blockchain and AI-enhanced trust management system	Dynamic trust evaluation using machine learning and blockchain	High computational resource demand for AI and blockchain integration
Sun et al. [40]	2022	Blockchain-enabled secure data sharing in VANETs	Improved trustworthiness of shared data using blockchain immutability	Increased latency in trust validation for real-time communication
Huang et al. [41]	2023	Proof of Stake (PoS)-based trust management in VANETs	Low energy consumption and faster consensus process	Vulnerability to stake accumulation attacks and collusion
Liu et al. [4]	2024	Smart contract-based automated trust management	Automated trust score updates with tamper-proof records	High computational cost and delays due to smart contract execution
Zhang et al. [28]	2023	Privacy-focused blockchain trust management with zero-knowledge proofs	Enhanced privacy and secure trust evaluation without revealing identities	Computationally expensive and slow cryptographic operations

"AT the end, we would like to thank the referees and editors for evaluating our manuscript. We have tried to address all recommendations of the reviewers, their concerns in a proper way and believe that our paper has improved considerably."
