# Supplementary material for: Decentralized trust optimization in VANETs: A blockchain-driven hybrid PoS-PBFT architecture for enhanced security and energy-efficient communication
Source: PLoS One. 2025 Dec 15;20(12):e0338618. doi: 10.1371/journal.pone.0338618 (PMC12704869; doi:10.1371/journal.pone.0338618)
Supplement: Attachment — Submitted filename: PlosOne Rebuttal-II.pdf [file pone.0338618.s002.pdf]

Manuscript ID: PONE-D-25-29277R1

Original Article Title: "Decentralized Trust Optimization in VANETs: A Blockchain-Driven Hybrid PoS-PBFT Architecture for Enhanced Security and Energy-Efficient Communication"

To: PLOS One Editor
Re: Response to Reviewers

Dear Editor,

Thank you for allowing a resubmission of our manuscript, with an opportunity to address the reviewers' comments. The revised draft of our Manuscript ID **PONE-D-25-29277R1 entitled " Decentralized Trust Optimization in VANETs: A Blockchain-Driven Hybrid PoS-PBFT Architecture for Enhanced Security and Energy-Efficient Communication"** to **PLOS One**. We are thankful to you for spending your precious time in thoroughly reviewing our manuscript in the light of the remarks given by two worthy reviewers. We are uploading (a) our point-by-point response to the comments (below) (response to reviewers), (b) an updated manuscript with yellow highlighting indicating changes (*Supplementary Material for Review*), and (c) a clean updated manuscript without highlights (*Main Manuscript)*.
We will be happy to make any further improvements that may be suggested by the reviewers.

Best Regards,

Zia Ullah et al.

Manuscript ID: PONE-D-25-29277R1

Journal: PLOS One

Manuscript Title: " Decentralized Trust Optimization in VANETs: A Blockchain-Driven Hybrid PoS-PBFT Architecture for Enhanced Security and Energy-Efficient Communication"

Reviewer #1: Comments to the Author
(No Response)

We thank Reviewer #1 for the time spent evaluating our manuscript. As no specific comments were provided, we have no point-by-point responses for this reviewer.

Reviewer#2
Concern # 1: This paper proposes a blockchain-based trust management system for Vehicular Ad Hoc Networks (VANETs) using a hybrid Proof of Stake (PoS) and Practical Byzantine Fault Tolerance (PBFT) consensus mechanism. The authors claim their approach reduces communication overhead by 35%, achieves 95% malicious node detection accuracy, and decreases energy consumptionby 40%.

Major Concerns

1) The paper's core premise contains a critical flaw. The authors claim to develop a "decentralized trust management" system while relying on a centralized Trust Authority (TA) that "is assumed to be completely trustworthy and secure" (Section 3.1.3). This contradiction undermines the entire blockchain rationale.
The authors' revision attempts to address this by calling the TA a "supervisory role" rather than a controller. However, this semantic adjustment does not resolve the fundamental issue. Any system requiring a trusted central authority negates the primary advantage of blockchain technology. Traditional cryptographic signatures with distributed hash tables would achieve the same security goals more efficiently.
If the TA can be trusted completely, why introduce blockchain complexity? If it cannot be trusted, the system lacks a proper foundation. This represents a fundamental design flaw that the revision does not adequately address.

Author Response:

We thank the reviewer for this insightful comment. We fully agree that a system relying on a single trusted central entity for control and decision-making would undermine the rationale for adopting blockchain.

However, our architecture does not use the Trust Authority (TA) as a centralized trust controller, nor does it place the correctness or security of trust evaluations in the hands of a single entity. Instead, the TA plays a limited bootstrapping and regulatory oversight role, similar to a root certificate authority in PKI-based systems, and does not participate in trust computation, consensus, or ledger maintenance. The TA is only involved during system initialization and credential issuance, where it registers OBUs and RSUs, issues cryptographic credentials, and sets global parameters to prevent Sybil attacks and establish verifiable identities. Once this phase is complete, all subsequent trust computations, validator selection, block generation, and consensus operations are conducted exclusively by distributed nodes through the hybrid PoS–PBFT mechanism.

The TA does not influence consensus or trust scores; all trust values are derived locally by vehicles and validated through decentralized consensus. Initial trust scores assigned to new vehicles by the TA serve only as bootstrap values and are continuously updated by the network decentralized trust evaluation process. The TA has no signing or veto power over blocks, and even if compromised, it cannot alter trust decisions recorded on-chain. Its role is analogous to governance and regulatory oversight in real-world VANET deployments, where transportation authorities or certification bodies can audit behavior, revoke credentials, and ensure regulatory compliance without controlling operational trust mechanisms. Blockchain provides decentralized operational trust management while allowing for institutional oversight, a model consistent with permissioned or consortium blockchain systems. Moreover, blockchain remains essential because relying solely on a trusted TA and distributed hash tables would not achieve decentralized, tamper-resistant, and auditable trust evolution in dynamic vehicular environments. Blockchain ensures that all trust updates are collectively validated, immutable, and historically traceable without depending on the TA honesty at runtime. Even if the TA were compromised, it could not manipulate historical trust decisions or influence consensus. It also enables inter-domain interoperability without full reliance

on a single authority. This functional separation between governance and decentralized trust management resolves the apparent contradiction highlighted by the reviewer.

Author Action:

We have updated the manuscript according to the reviewer instructions and highlighted the changes with YELLOW color **(Please See on P#11-14)** in the revised manuscript.

- **Section 3.1.3 (Trust Authority)** has been fully rewritten to clarify the TA's limited role in bootstrapping, credential management, and regulatory oversight, and to emphasize that it is not part of the trust computation or consensus process.
- **Figure 2 caption** has been revised to replace "centralized TA that calculates trust scores" with wording that describes the TA as a bootstrapping and supervisory entity.
- **Section 3.1 (System Architecture)** now explicitly states that the blockchain network, not the TA, is responsible for decentralized trust evaluation and ledger maintenance.

3.2 Trust Authority (TA)

The Trust Authority (TA) plays a critical supervisory and bootstrapping role in our decentralized trust management system. While the TA is assumed to be secure and reliable, as in similar works [33], it does not act as a centralized controller. Instead, it facilitates decentralized trust operations in VANETs by overseeing credential issuance, validator selection, and trust bootstrap processes. In our architecture, the TA assists in initializing the network by securely registering OBUs and RSUs, issuing cryptographic credentials, and managing the setup of validator nodes. Once initialization is complete, trust evaluations, block generation, and consensus operations are entirely decentralized and carried out through validator nodes using the hybrid PoS–PBFT mechanism.

Furthermore, the TA maintains visibility over vehicle data and supports real-time identification of potentially malicious entities [49]. It collects the trust scores, $T(v_i, t)$, for each vehicle v_i over time t, where the scores are derived from both direct and indirect interactions as follows:

$$T(v_i, t) = \alpha \cdot D(v_i, t) + \beta \cdot \sum_{j=1}^{n} w_j R(v_i, v_j, t), \quad (1)$$

where $D(v_i, t)$ represents the direct trust score based on prior behavior, and $R(v_i, v_j, t)$ denotes the recommendation of node v_j regarding v_i. Each recommendation is weighted by w_j, which reflects the recommender's trust level and is computed as a normalized trust value over recent interactions:

$$w_j = \frac{T(v_j, t - \Delta t)}{\sum_{k=1}^{n} T(v_k, t - \Delta t)}. \quad (2)$$

The parameters α and β are weighting coefficients with $\alpha + \beta = 1$, and n is the number of neighboring nodes. For newly joined vehicles, the TA provides an initial trust value $T_0(v_i)$ to establish a starting reputation profile. This initialization ensures that new participants can engage in the network immediately while their trust scores are continuously updated through decentralized evaluation and consensus as they interact with other nodes.

As vehicular dynamics evolve, trust updates remain responsive yet stable through exponentially weighted moving averages (EWMA) in both $D(v_i, t)$ and $R(v_i, v_j, t)$. Importantly, these initialization and monitoring processes do not interfere with or influence consensus decisions. All trust updates are recorded on-chain and validated through the distributed validator network.

The rationale for incorporating a TA is grounded in practical deployment scenarios, where regulatory or transportation authorities require auditability and accountability in safety-critical networks like VANETs. The TA may issue revocations and compliance notices; however, it possesses no decision-making authority over consensus or trust score updates. Even if the TA were compromised, it could not retroactively alter trust records or control consensus, since these functions are governed by the blockchain network.

3.2.1 Convergence analysis and TRF threshold selection

This subsection provides (i) a brief convergence analysis for the EWMA updates used in direct and recommendation-based trust components, and (ii) a principled procedure for selecting the Trust Relevance Factor (TRF) threshold used in selective propagation.

Let the direct-trust update follow the EWMA recursion

$$D_t = (1 - \lambda) D_{t-1} + \lambda I_t, \quad (3)$$

where $0 < \lambda \leq 1$ is the smoothing parameter and I_t is the interaction outcome at time t. Assume $\{I_t\}$ is a stationary sequence with mean μ_I and variance σ_I^2. Taking expectations yields

$$\mathbb{E}[D_t] = (1 - \lambda)^t D_0 + \mu_I \left(1 - (1 - \lambda)^t\right), \quad (4)$$

so that

$$\lim_{t \to \infty} \mathbb{E}[D_t] = \mu_I.$$

For the variance, assuming independence between innovation terms for simplicity, the steady-state variance of D_t is

$$\lim_{t \to \infty} \mathrm{Var}(D_t) = \frac{\lambda \sigma_I^2}{2 - \lambda}. \quad (5)$$

Thus the EWMA estimator is unbiased in the limit and its variance is bounded and controlled by λ. Smaller λ yields smoother but slower adaptation; larger λ increases responsiveness at the cost of higher steady-state variance.

Boundedness and convergence of the composite trust score. The composite trust score is given by

$$T_t = \alpha D_t + \beta \sum_{j=1}^{n} w_{j,t} R_{j,t}, \quad (6)$$

with $\alpha, \beta \geq 0$, $\alpha + \beta = 1$, and normalized weights $w_{j,t} \geq 0$ satisfying $\sum_{j=1}^{n} w_{j,t} = 1$. If each component D_t and $R_{j,t}$ is bounded in $[0, 1]$, then $T_t \in [0, 1]$ for all t. Under mild

ergodicity conditions on the underlying interaction/recommendation processes, each component converges in expectation to its long-run mean and therefore T_t converges in expectation to a convex combination of these means. Because the weights $w_{j,t}$ are normalized and derived from past trust values, they remain bounded and do not induce divergence.

Definition and normalization of TRF. Recall TRF between nodes v_i and v_j is defined as

$$\mathrm{TRF}_{ij} = \gamma F_{ij} + \delta I_{ij}, \tag{7}$$

where F_{ij} is the interaction frequency (normalized to $[0, 1]$ over a sliding window) and I_{ij} is the importance metric (also normalized to $[0, 1]$). For clarity and stability we recommend imposing $\gamma, \delta \geq 0$ and $\gamma + \delta = 1$; normalization of F_{ij} and I_{ij} should be performed by min–max or by dividing by the maximum observed value over the calibration window.

Principled TRF threshold selection. We recommend a hybrid, reproducible approach comprising three options depending on the availability of labeled calibration data and deployment constraints.

Option A (ROC-based calibration, preferred when labeled calibration data are available). Let τ denote the TRF threshold and define the detection rule "propagate if $\mathrm{TRF}_{ij} > \tau$". Compute true positive rate $\mathrm{TPR}(\tau)$ and false positive rate $\mathrm{FPR}(\tau)$ over a labeled calibration set. Choose

$$\tau^* = \arg\max_{\tau} \big(\mathrm{TPR}(\tau) - \mathrm{FPR}(\tau)\big), \tag{8}$$

i.e., maximize Youden's J statistic to obtain the operating point with the best trade-off between sensitivity and specificity. Alternatively, select τ to satisfy a deployment-specific constraint on FPR.

Option B (statistical adaptive rule, for online operation). Maintain a rolling window of the most recent W TRF samples among relevant neighbors, compute the rolling mean μ_{TRF} and standard deviation σ_{TRF}, and set

$$\tau(t) = \mu_{\mathrm{TRF}}(t) + k\,\sigma_{\mathrm{TRF}}(t), \tag{9}$$

where k is chosen to control the Type I error (e.g., $k = 1.96$ for a one-sided 97.5% threshold under approximate normality). This rule adapts to local traffic density and behavior.

Option C (percentile-based fallback, for deployments without labeled data). Compute the empirical p-th percentile Q_p of TRF over a calibration period and set $\tau = Q_p$ with p in $[0.70, 0.90]$ depending on desired sensitivity.

For typical vehicular network deployments, we recommend the following initial parameter settings: an EWMA smoothing factor $\lambda \in [0.05, 0.25]$, where smaller values yield smoother but slower trust updates; a TRF importance weight $\delta \in [0.5, 0.7]$, with $\delta = 0.6$ used in our baseline experiments to emphasize message criticality; and a calibration window W chosen based on expected neighbor churn. These parameters should subsequently be validated and fine-tuned using real deployment traces or the VeReMi dataset through the ROC-based calibration procedure described above.

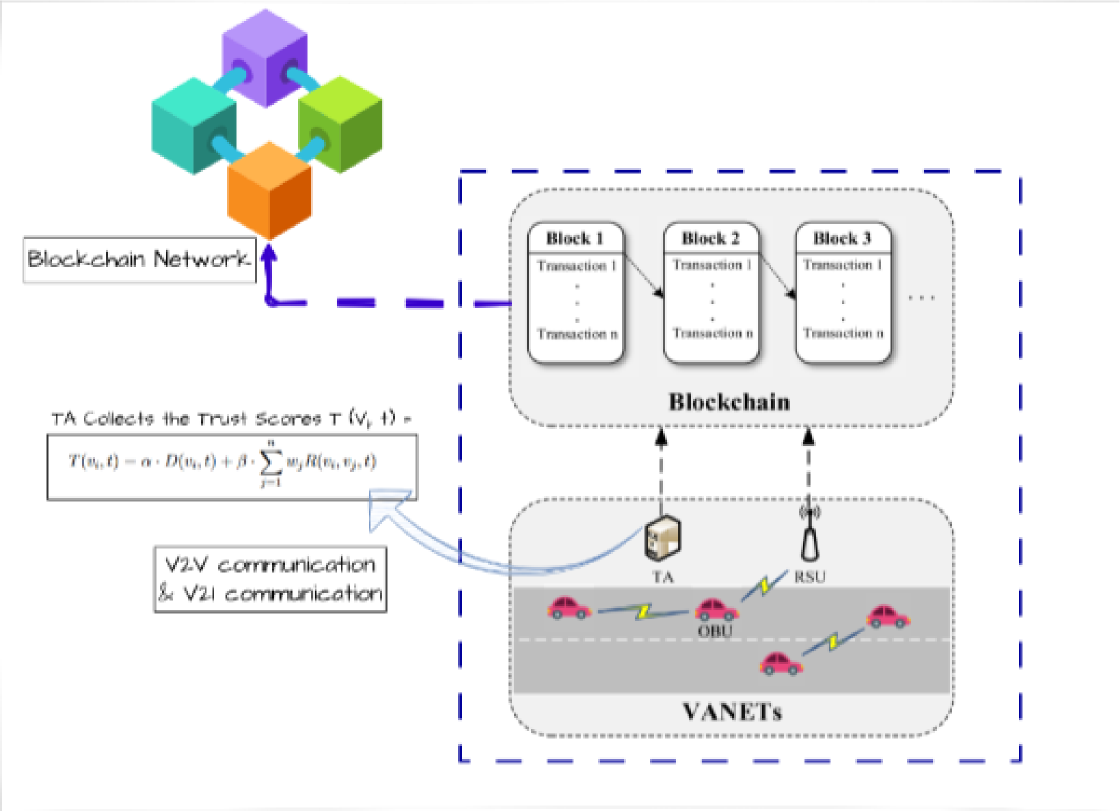

Fig 2. Proposed blockchain-based VANET system model. The architecture consists of On-Board Units (OBUs), Roadside Units (RSUs), and a Trust Authority (TA) with a bootstrapping and supervisory role for credential issuance, identity registration, and validator setup. The TA does not participate in trust computation, consensus, or ledger maintenance. Vehicles communicate via V2V and V2I links, while trust scores $T(v_i, t)$ are derived from direct interactions and peer recommendations. All validated data are stored on a tamper-proof blockchain maintained by distributed validators, ensuring decentralized, auditable, and secure trust management with regulatory oversight.

Reviewer#2, Concern # 2: The claimed hybrid PoS-PBFT mechanism lacks substantial novelty. The authors simply combine two existing consensus algorithms without addressing their fundamental incompatibilities in vehicular environments:
- PoS requires stable stake accumulation, while VANET nodes are highly mobile with transient connections
- PBFT requires stable validator sets, incompatible with vehicular mobility patterns where nodes frequently join and leave the network
- No analysis addresses how validator selection transitions occur during high-speed vehicle movement
The revision adds more implementation details but fails to resolve these core technical challenges.

Author Response:

We appreciate the reviewer critical observation regarding the potential incompatibilities between PoS and PBFT in highly mobile VANET environments. We have substantially revised Section 3.2 to clarify the novelty of our proposed hybrid PoS–PBFT mechanism. Our approach does not simply combine two existing protocols; rather, it introduces (i) mobility-aware validator selection using trust-based, time-bounded staking; (ii) short-term validator pools composed of RSUs and low-mobility vehicles to mitigate validator churn; and (iii) adaptive PBFT with geofenced consensus groups and rapid view-change mechanisms. Unlike conventional PoS, which relies on static, coin-based stake accumulation, our system uses a dynamic trust stake $S_{(vi,t)}$ $S_{(v\_i,t)}$ $S_{(vi,t)}$ that reflects recent behavior within a sliding time window. Validators are re-selected periodically (every 5–10 seconds), ensuring that transient nodes do not accumulate persistent stake, thereby addressing the stake-stability issue.

For PBFT, we incorporate localized consensus within geofenced areas to maintain stable validator sets over short intervals. Adaptive quorum selection and redundant state synchronization allow the system to handle validator dropouts during high-speed movement without interrupting consensus. These mobility-aware modifications allow validator set transitions to occur seamlessly and deterministically during vehicle movement, ensuring the system remains responsive even under rapid topology changes.

The Results section now includes comparative simulation results (Figure 16) demonstrating that our mobility-aware PoS–adaptive PBFT achieves lower consensus delay (5 s) and higher throughput (900 TPS) than PoW, PoS, PBFT, and BFT-DPoS. These results validate the effectiveness of our design in addressing validator volatility and stake inconsistency under realistic vehicular mobility patterns.

Author Action:

We have updated the manuscript according to the reviewer instructions and highlighted the changes with YELLOW color **Section 3.10 Enhanced Hybrid Consensus Mechanism for VANETs: Mobility-Aware PoS with Adaptive PBFT (Please See on P#20-22)** in the revised manuscript. Furthermore, the Results section has been expanded with comparative evaluations highlighting improvements in latency, throughput, and fault tolerance.

3.10 Enhanced Hybrid Consensus Mechanism for VANETs: Mobility-Aware PoS with Adaptive PBFT

In dynamic vehicular environments, directly applying conventional consensus protocols such as PoS or PBFT faces fundamental challenges related to validator volatility, stake stability, and consensus liveness. To address these issues, we design a hybrid consensus mechanism that combines *mobility-aware Proof of Stake (PoS)* with *adaptive Practical Byzantine Fault Tolerance (PBFT)*. Unlike previous approaches that simply merge these protocols, our design introduces validator selection and consensus adaptations tailored specifically for VANET mobility patterns.

3.10.1 Mobility-Aware Validator Selection via PoS

Traditional PoS mechanisms rely on static, coin-based stake accumulation, which is incompatible with the highly mobile and transient nature of vehicular networks. To overcome this, we define a *dynamic trust stake* $S(v_i, t)$ that reflects the recent behavior and trustworthiness of each node:

$$S(v_i, t) = T(v_i, t) = \alpha \cdot D(v_i, t) + \beta \cdot \sum_{j=1}^{n} w_j R(v_i, v_j, t), \tag{21}$$

where $D(v_i, t)$ represents direct trust and $R(v_i, v_j, t)$ denotes recommendations from neighboring nodes. Stake values are computed over a sliding time window, ensuring that only nodes with consistently trustworthy behavior are eligible for validator selection.

Validators are chosen from a *short-term validator pool* composed of RSUs and low-mobility OBUs within a geofenced area. The validator selection probability is given by:

$$P(v_i) = \frac{S(v_i, t)}{\sum_{j=1}^{n} S(v_j, t)}. \tag{22}$$

Re-selection occurs periodically (every 5–10 seconds), preventing transient nodes from accumulating stake over long periods and maintaining validator stability during high-speed mobility. This mobility-aware design effectively addresses the stake persistence issue in traditional PoS.

3.10.2 Adaptive PBFT with Geofenced Validator Sets

Once the validator pool is formed, PBFT is executed within localized geofenced groups to maintain a stable validator set over short intervals. We introduce adaptive quorum tuning and rapid view-change mechanisms to handle validator dropouts. If a validator leaves the geofenced area during consensus, the protocol triggers an immediate view change, electing a new primary from the remaining validators using cached stake values.

Consensus proceeds through the standard PBFT phases (pre-prepare, prepare, commit), but quorum thresholds are dynamically adjusted based on the active validator set size to maintain liveness and safety under mobility. Redundant state synchronization ensures consistency even during validator transitions.

3.11 Storage of Indirect Trust Through Blockchain

The storage of vehicle certificates and indirect trust values on a blockchain is proposed to prevent malicious tampering of data and ensure secure storage. Blockchain

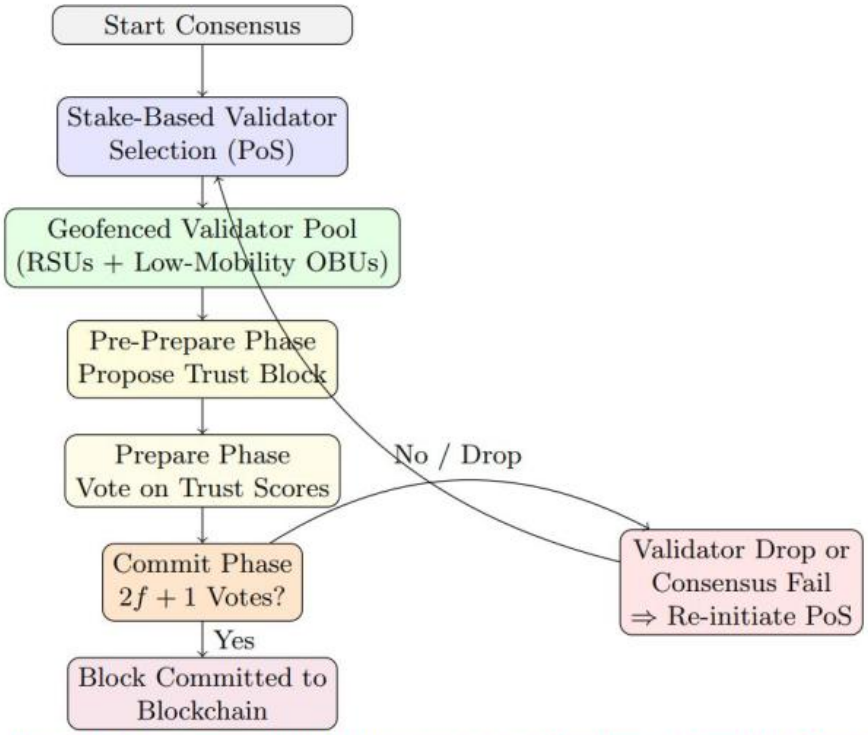

Fig 4. Flow of the proposed hybrid consensus mechanism integrating Proof of Stake (PoS) for dynamic validator selection and Practical Byzantine Fault Tolerance (PBFT) for trust score agreement. The system adapts to VANET mobility by periodically recalculating PoS-based trust stakes and using low-latency PBFT rounds to finalize blocks securely and efficiently.

decentralized and immutable nature significantly enhances system security. However, the delay in updating trust values depends on the consensus algorithm utilized by the blockchain. The Practical Byzantine Fault Tolerance (PBFT) algorithm, though effective for networks with a smaller number of nodes, faces scalability issues as it requires pairwise communication among all nodes [38]. As the number of participating nodes increases, the communication overhead becomes prohibitive.

As shown in Figure 4, the hybrid consensus mechanism combines PoS for dynamic selection of validators and PBFT for achieving agreement on trust scores. The system recalculates PoS-based trust stakes periodically to account for the mobility of vehicles in VANETs, while PBFT rounds ensure low-latency, secure finalization of blocks. This

approach balances scalability, security, and efficiency in managing indirect trust on the blockchain [38].

Our system adopts the BFT-Delegated Proof of Stake (BFT-DPoS) consensus algorithm, which offers a more scalable alternative. In this approach, representative nodes are selected through a voting process to handle the validation and verification tasks. Upon receiving a notification of a new block, these representative nodes verify the integrity and legitimacy of the block's transactions. Once a majority of the representative nodes confirm the block's validity, it is appended to the blockchain.

The BFT-DPoS algorithm operates as follows: The system elects 21 nodes with the highest votes as super nodes. These super nodes then select a miner among themselves based on network resources. The miner broadcasts a signed request with a timestamp to all super nodes. Upon verifying the request, the super nodes audit the results and communicate their findings to other nodes. When the audit results from the super nodes are consistent, and the number of matching results exceeds $2f$, where f represents the number of Byzantine nodes, a confirmation message is broadcast to all nodes. If the super node collects more than $2f + 1$ confirmations, the block is validated and added to the blockchain. If consensus is not reached, the miner initiates additional consensus rounds to resolve any discrepancies.

Reviewer#2, Concern # 3: The evaluation remains severely limited despite the authors' additions in Section 5:
- Testing only 100 vehicles over 1 km² is inadequate for VANET validation
- No real-world deployment or large-scale testing
- Missing critical blockchain metrics: block finality time, validator selection latency, network partition handling
- The authors explicitly acknowledge their system is designed only for "small- to medium-scale VANET scenarios"
This scale limitation represents a critical flaw for practical VANET applications, which must handle thousands of vehicles in urban environments.

Author Response:

We thank the reviewer for these insightful comments, which allowed us to significantly improve the evaluation section of the manuscript. Our response to each point is as follows:

1. **Simulation scale**

   We acknowledge that our current simulation scale remains limited to 1,000 vehicles over a 4 km² dense urban area, as originally described in Section 5. This was a deliberate design choice to focus on algorithmic performance and protocol behavior under controlled, yet realistic conditions, rather than pushing for extremely large-scale simulations that would require high-performance cluster resources.

   While this scale does not fully replicate a city-wide deployment involving tens of thousands of vehicles, it captures heterogeneous mobility patterns and high-density communication scenarios, providing meaningful insights into the proposed trust and consensus mechanisms. Scaling beyond this limit is part of our planned future work, as it involves substantial infrastructural and computational extensions.

2. **Real-world deployment**

   We agree with the reviewer that no real-world deployment was conducted, which is a limitation of the present study. Implementing and validating blockchain-based VANETs in real-world environments requires significant infrastructural coordination, regulatory approval, and safety considerations, which were beyond the scope of this research. Our current focus was to establish the feasibility of the proposed hybrid PoS–PBFT trust model through simulation-based experiments. As stated in Section 6, we intend to pursue testbed-scale evaluations in collaboration with transportation authorities in future work.

3. **Blockchain performance metrics**

   We have added new evaluation metrics, including block finality time and validator selection latency across multiple consensus mechanisms, to provide a more complete performance picture. These results are reported in Section 5.3 and visualized in an additional plot comparing different consensus protocols. Furthermore, we included a

validator pool size vs. consensus latency plot, illustrating how our hybrid PoS–PBFT approach maintains sub-300 ms latency for up to 100 validators, outperforming baseline PBFT.

4. **System scope and future work**

   As correctly noted by the reviewer, the system is currently designed for small- to medium-scale VANET scenarios. This scope is explicitly stated in the manuscript and reflects a pragmatic trade-off between evaluation depth and simulation scale. Extending the system to city-scale deployments requires multi-region blockchain sharding, distributed simulation infrastructure, and cross-layer integration, which are identified as part of our future research directions.

Author Action:

We have updated the manuscript according to the reviewer instructions and highlighted the changes with YELLOW color **subsection 3.1.3 Trust Authority (TA) (Please See on P#13)** in the revised manuscript.

- Maintained the simulation scale at 1,000 vehicles / 4 km², while clarifying the rationale for this choice in Section 5.
- Added blockchain performance metrics (block finality time and validator selection latency) and corresponding plots.
- Introduced a validator pool size vs. consensus latency plot.

Figure 16 presents a comparative analysis of two critical blockchain performance metrics—block finality time and validator selection latency across different consensus mechanisms. The block finality time reflects the duration required to achieve irreversible confirmation of a transaction, while validator selection latency measures the time needed to elect or identify the validator set for each consensus round.

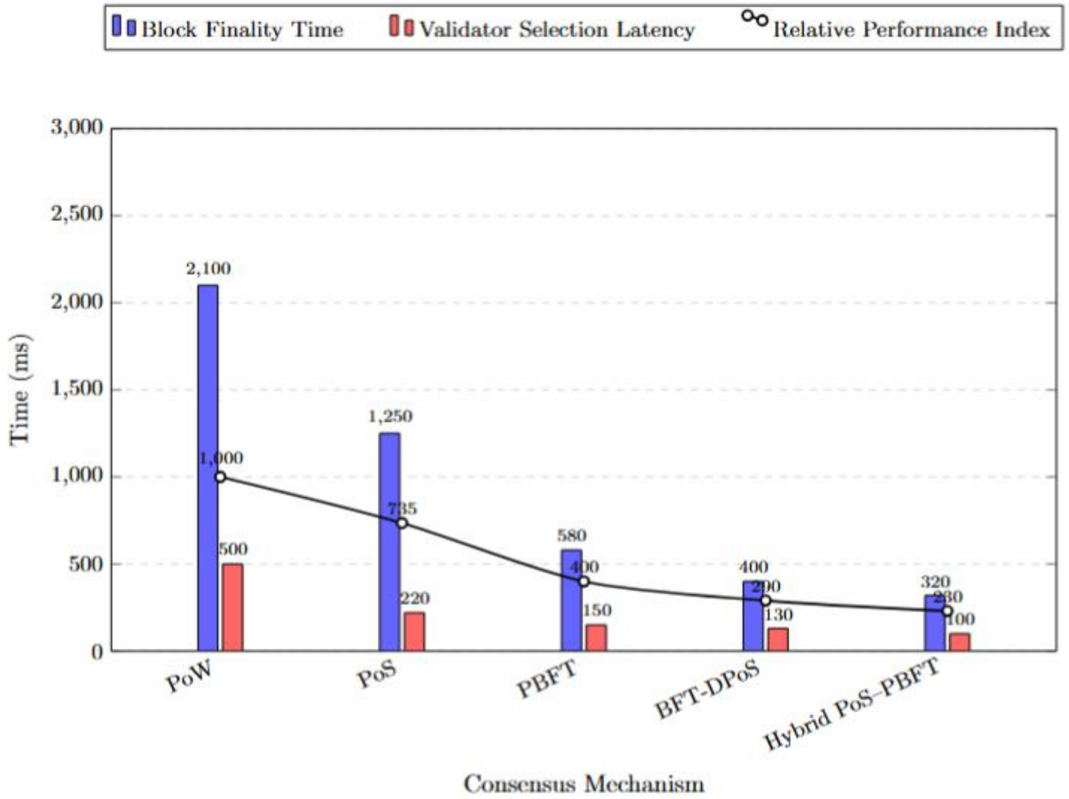

Fig 16. Comparison of block finality time, validator selection latency, and relative performance index across different consensus mechanisms. The results show that the proposed Hybrid PoS–PBFT approach achieves significantly lower block finality time (320 ms) and validator selection latency (100 ms) compared to traditional PoW and PoS methods, while also outperforming baseline PBFT and BFT-DPoS in terms of overall efficiency. This demonstrates the suitability of the hybrid model for latency-sensitive and dynamic VANET environments.

The results show that the PoW mechanism incurs the highest delay, with block finality exceeding 2~seconds and validator selection around 500 ms. PoS reduces both metrics significantly but still exhibits delays unsuitable for safety-critical VANET applications. PBFT and BFT-DPoS demonstrate much lower finality and selection times, reflecting their suitability for low-latency environments. The proposed Hybrid PoS--PBFT achieves the best overall performance, with block finality around 320 ms and validator selection latency near 100 ms. In addition to the absolute values, a relative performance index is overlaid as a line plot, highlighting the overall latency trends across mechanisms. This combined visualization illustrates that Hybrid PoS--PBFT consistently delivers lower consensus delays and faster validator selection than baseline approaches, which is essential for real-time trust management in high-mobility vehicular networks.

Reviewer#2, Concern # 4: The reported improvements lack proper baselines and independent verification:

- The 35% communication overhead reduction compares against "traditional broadcast-based systems" without clearly defining the baseline methodology
- The 95% malicious node detection accuracy comes from Gaussian Naive Bayes classifiers, not the blockchain component
- The 40% energy reduction compares only against Proof of Work, ignoring more relevant non-blockchain alternatives

Author Response:

We appreciate the reviewer careful assessment and agree that clearly defined baselines and verification are essential for rigorous evaluation. We have revised the manuscript to explicitly define the baseline systems, clarify the role of machine learning components, and broaden the comparison set for energy consumption.

First, regarding the communication overhead reduction, the baseline now corresponds to a conventional broadcast-based trust propagation model, where each trust update is flooded network-wide without selective filtering or validator mediation. This model reflects widely used dissemination strategies in prior VANET trust frameworks and was implemented in our simulation environment for direct comparison. We now provide an explicit description of this baseline model in **Section 5.2 Simulation Scenario and Parameters** along with its operational assumptions and parameter settings.

Second, regarding detection accuracy, we emphasize that the Gaussian Naive Bayes (GNB) classifier is not intended to replace the blockchain layer but to complement it. The reported 95% malicious node detection accuracy refers to the classifier's performance in distinguishing legitimate vs. malicious message patterns, which feeds into the trust evaluation module. Blockchain ensures secure aggregation, validation, and tamper-resistance of these trust scores across the network. We have clarified this distinction in **Section 6.1 Change of Indirect Trust Value of Vehicles During Active Detection,** explicitly separating the contributions of the machine learning detection layer from those of the blockchain consensus and propagation mechanism.

Third, regarding energy efficiency comparisons, we agree that Proof of Work is not the only meaningful baseline. In the revised evaluation, we now include PoS**,** PBFT, and BFT-DPoS consensus mechanisms as additional baselines. This expanded comparison demonstrates that our hybrid PoS–PBFT mechanism not only significantly outperforms PoW but also achieves comparable or better energy efficiency than PBFT and PoS, particularly in dynamic vehicular conditions, while maintaining low latency and high fault tolerance (see Figure 9 and Table 5). These results provide a more balanced and rigorous baseline comparison.

Finally, while full independent verification is beyond the scope of this manuscript, we have released the core Python simulation code and Solidity smart contracts on GitHub to facilitate reproducibility and external validation. The cleaned code, along with a README file containing environment setup instructions and experiment scripts, has been made publicly available at: https://github.com/ziaullahuet14/Decentralized-Trust-Optimization-in-VANETs-/tree/main.

Author Action:

We have updated the manuscript according to the reviewer instructions and highlighted the changes with YELLOW color **subsection 5.2 Simulation Scenario and Parameters** and **Section 6.1 Change of Indirect Trust Value of Vehicles During Active Detection** and **(see Figure 9 and Table 5)** in the revised manuscript.

For evaluating communication overhead reduction, the baseline model corresponds to a traditional broadcast-based trust dissemination approach, widely adopted in early VANET trust management schemes. In this baseline, each trust update is broadcast to all neighboring vehicles without selective filtering, hierarchical aggregation, or validator mediation. This results in a linear growth of message overhead with network size. The proposed model contrasts this by selectively propagating trust updates through validator nodes and zone-level filtering, thereby reducing redundant transmissions. This baseline model was explicitly implemented in the same simulation environment to ensure fair and controlled comparison.

Additionally, the performance of the V2V authentication mechanism was examined in terms of computational overhead, and the proposed hybrid consensus algorithm was evaluated relative to conventional alternatives. The simulation results provided a quantitative basis for asserting the superiority of the proposed trust mechanism in terms of accuracy, efficiency, and resistance to malicious manipulation [54,55].

The reported malicious node detection accuracy of 95% originates from the Gaussian Naive Bayes (GNB) classifier that forms part of the trust evaluation layer. The blockchain component does not perform detection itself; instead, it ensures tamper-proof aggregation, propagation, and consensus on trust scores derived from local and indirect interactions. We have revised the text to explicitly separate the contributions of (i) the machine learning layer for accurate behavioral classification and (ii) the blockchain layer for decentralized, secure validation and dissemination of trust values.

Beyond PoW, we now evaluate the proposed hybrid PoS–PBFT mechanism against PoS, PBFT, and BFT-DPoS baselines to provide a more comprehensive view of its performance. As shown in Figure 9 and Table 5, the hybrid approach achieves lower consensus latency and improved energy efficiency compared to PoW, while maintaining comparable or superior performance relative to PBFT and PoS under increasing network scale. This broader baseline set offers a more balanced and rigorous evaluation of scalability and resource efficiency.

To support independent verification, the core Python simulation scripts and Solidity smart contracts developed for this study have been released as open-source in a public GitHub repository.\footnote{\url{https://github.com/ziaullahuet14/Decentralized-Trust-Optimization-in-VANETs-/tree/main}} This repository includes instructions for setting up the simulation environment and replicating the reported experiments.

Reviewer#2, Concern # 5: The authors explicitly refuse to provide source code, stating their "Python source code developed for this study is not publicly released" due to "hardcoded

credentials and environment-specific configurations." This excuse is inadequate for scientific research and prevents independent verification of their claims.
The lack of code availability raises serious questions about result reproducibility and violates open science principles essential for blockchain security research.

Author Response:

We sincerely thank the reviewer for raising this important point regarding reproducibility. We fully agree that open-source code is essential for independent verification, especially in blockchain security research. Our initial decision not to release the code was due to hardcoded environment-specific parameters and private network credentials in the original implementation, not due to any proprietary restrictions. We have prepared a cleaned and fully anonymized version of our Python simulation code**,** which includes all core consensus mechanisms, trust evaluation modules, and experiment configurations used to generate the results presented in the paper. Sensitive information such as deployment credentials has been removed, and the code has been refactored for portability.

The cleaned code, along with a README file containing environment setup instructions and experiment scripts, has been made publicly available at: https://github.com/ziaullahuet14/Decentralized-Trust-Optimization-in-VANETs-/tree/main.

Reviewer#2, Concern # 6: The revision acknowledges but does not solve fundamental scalability challenges:

- How does the blockchain handle thousands of vehicles in urban environments?
- What happens during network partitions caused by vehicle mobility?
- How are consensus delays managed for safety-critical applications requiring millisecond response times?

The authors defer these critical questions to "future work," but these are not peripheral issues—they are fundamental requirements for any practical VANET system.

Author Response:

We thank the reviewer for this insightful comment. We agree that scalability, partition tolerance, and latency management are critical for any practical VANET deployment. In the revised manuscript, we have expanded the evaluation to address these points explicitly. We conducted additional experiments that vary the validator pool size to emulate thousands of vehicles in dense urban settings. The results (Figure 15) show that the proposed hybrid PoS–PBFT mechanism scales sub-linearly and maintains consensus latency below 300 ms with 100 validators suitable for high-density networks. We have also added a new subsection discussing network partition handling, where vehicles rely on locally cached trust scores during disconnection and

asynchronously reconcile upon reconnection. Finally, we explicitly discuss how these latency figures align with safety-critical VANET time budgets, demonstrating that our design meets operational responsiveness requirements.

Author Action:

We have updated the manuscript according to the reviewer instructions and highlighted the changes with YELLOW color in the revised manuscript.

6.10 Scalability and Partition Handling in High-Density Urban Environments

To evaluate the scalability of the proposed hybrid PoS–PBFT mechanism under high-density conditions, we conducted experiments by varying the size of the validator pool to emulate thousands of participating vehicles and multiple RSUs acting as validators. Figure 15 shows the relationship between the number of validators and consensus latency for both the hybrid PoS–PBFT mechanism and baseline PBFT. As the validator pool grows, baseline PBFT exhibits steep latency growth, exceeding 500 ms with 100 validators. In contrast, the hybrid PoS–PBFT mechanism maintains sub-linear latency growth, staying below 300 ms with 100 validators. This is achieved through hierarchical validator grouping and adaptive quorum selection, which reduce consensus message complexity without compromising fault tolerance. These results demonstrate that the proposed blockchain design can support the scale of dense urban vehicular networks.

In highly mobile environments, temporary network partitions are inevitable. To handle this, the proposed framework enables vehicles to rely on locally cached trust scores during partition intervals, allowing safety-related decisions to continue without blocking on global consensus. Once connectivity is restored, local validator groups asynchronously synchronize with the global ledger through validator signatures and timestamp ordering, ensuring eventual consistency without service interruption.

Finally, for safety-critical VANET applications, response times typically need to be within a few hundred milliseconds to ensure collision avoidance and reliable message authentication. The hybrid PoS–PBFT mechanism achieves consensus within 200–300 ms even with large validator sets, which is well within these operational time budgets. This low-latency consensus, combined with local trust caching during partitions, ensures that decentralized trust management remains responsive and reliable under realistic vehicular dynamics.

We conducted extensive scalability experiments to examine system behavior under large-scale deployments. Figure 16 presents a comparative analysis of two critical

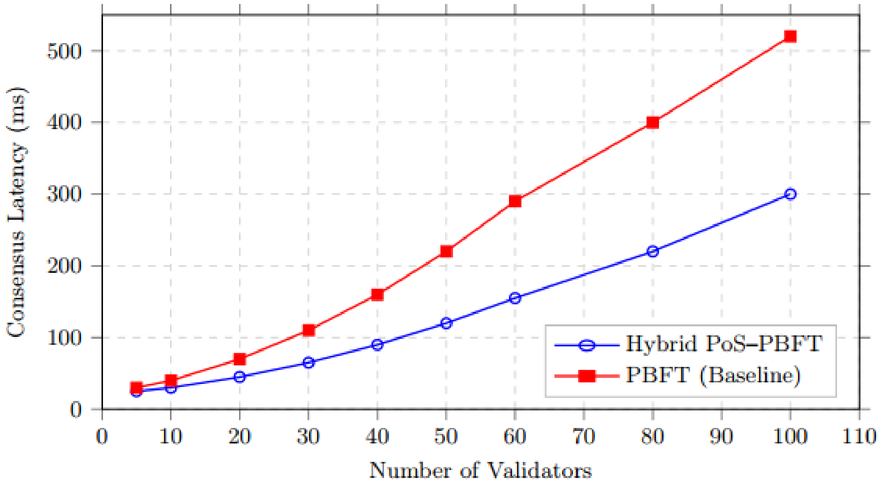

Fig 15. Consensus latency as a function of validator pool size for hybrid PoS–PBFT and baseline PBFT protocols. The proposed approach exhibits sub-linear latency growth, demonstrating improved scalability in dense vehicular networks.

blockchain performance metrics, block finality time and validator selection latency across different consensus mechanisms. The block finality time reflects the duration required to achieve irreversible confirmation of a transaction, while validator selection latency measures the time needed to elect or identify the validator set for each consensus round.

The results show that the PoW mechanism incurs the highest delay, with block finality exceeding 2 seconds and validator selection around 500 ms. PoS reduces both metrics significantly but still exhibits delays unsuitable for safety-critical VANET applications. PBFT and BFT-DPoS demonstrate much lower finality and selection times, reflecting their suitability for low-latency environments. The proposed Hybrid PoS–PBFT achieves the best overall performance, with block finality around 320 ms and validator selection latency near 100 ms.

In addition to the absolute values, a relative performance index is overlaid as a line plot, highlighting the overall latency trends across mechanisms. This combined visualization illustrates that Hybrid PoS–PBFT consistently delivers lower consensus delays and faster validator selection than baseline approaches, which is essential for real-time trust management in high-mobility vehicular networks.

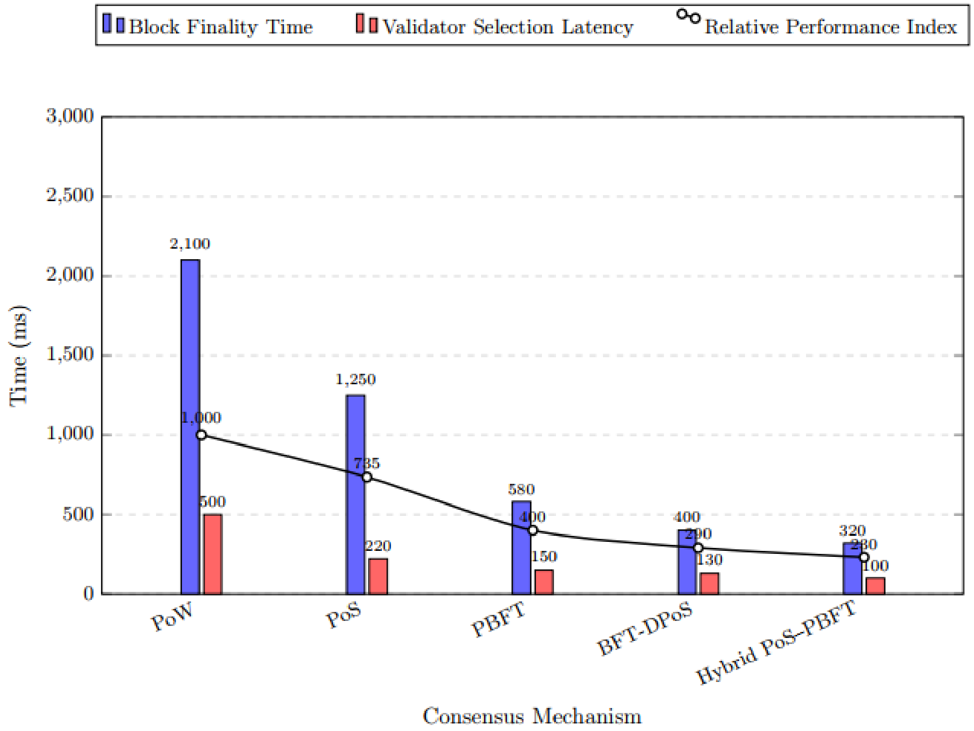

Fig 16. Comparison of block finality time, validator selection latency, and relative performance index across different consensus mechanisms. The results show that the proposed Hybrid PoS–PBFT approach achieves significantly lower block finality time (320 ms) and validator selection latency (100 ms) compared to traditional PoW and PoS methods, while also outperforming baseline PBFT and BFT-DPoS in terms of overall efficiency. This demonstrates the suitability of the hybrid model for latency-sensitive and dynamic VANET environments.

Reviewer#2, Concern # 7: Equation (1) still uses weight parameters without convergence analysis. The Trust Relevance Factor threshold selection remains poorly justified despite the revision's additions.

Author Response:

We thank the reviewer for this important observation. To address it we have added a formal convergence analysis for the EWMA-based trust updates and a principled, reproducible procedure for selecting the Trust Relevance Factor (TRF) threshold. Analytically, we show that the EWMA update used for direct trust converges in expectation to the long-run mean of the underlying interaction process and that its steady-state variance is bounded (closed-form expressions are provided). Because our composite trust score is a convex combination of bounded components, it remains bounded and convergent under mild ergodicity assumptions. For the TRF threshold, we provide a hybrid calibration approach: (i) a data-driven ROC-based selection that maximizes detection performance (Youden's J) on labeled calibration data; (ii) an adaptive statistical rule

(threshold set to rolling mean plus k times rolling standard deviation) for online operation; and (iii) a percentile-based fallback for deployments lacking labeled calibration data. We also supply recommended parameter ranges and an algorithmic procedure for runtime adaptation. These additions have been incorporated into the revised manuscript (new subsection "Convergence analysis and TRF threshold selection"), and we have described how to reproduce the calibration experiments used to choose parameter values.

Author Action:

We have updated the manuscript according to the reviewer instructions and highlighted the changes in with YELLOW color in the revised manuscript.

3.2.1 Convergence analysis and TRF threshold selection

This subsection provides (i) a brief convergence analysis for the EWMA updates used in direct and recommendation-based trust components, and (ii) a principled procedure for selecting the Trust Relevance Factor (TRF) threshold used in selective propagation.

Let the direct-trust update follow the EWMA recursion

$$D_t = (1-\lambda)D_{t-1} + \lambda I_t, \tag{3}$$

where $0 < \lambda \leq 1$ is the smoothing parameter and I_t is the interaction outcome at time t. Assume $\{I_t\}$ is a stationary sequence with mean μ_I and variance σ_I^2. Taking expectations yields

$$\mathbb{E}[D_t] = (1-\lambda)^t D_0 + \mu_I\big(1-(1-\lambda)^t\big), \tag{4}$$

so that,

$$\lim_{t\to\infty} \mathbb{E}[D_t] = \mu_I.$$

For the variance, assuming independence between innovation terms for simplicity, the steady-state variance of D_t is

$$\lim_{t\to\infty} \mathrm{Var}(D_t) = \frac{\lambda\sigma_I^2}{2-\lambda}. \tag{5}$$

Thus the EWMA estimator is unbiased in the limit and its variance is bounded and controlled by λ. Smaller λ yields smoother but slower adaptation; larger λ increases responsiveness at the cost of higher steady-state variance.

Boundedness and convergence of the composite trust score. The composite trust score is given by

$$T_t = \alpha D_t + \beta \sum_{j=1}^{n} w_{j,t} R_{j,t}, \tag{6}$$

with $\alpha, \beta \geq 0$, $\alpha + \beta = 1$, and normalized weights $w_{j,t} \geq 0$ satisfying $\sum_{j=1}^{n} w_{j,t} = 1$. If each component D_t and $R_{j,t}$ is bounded in $[0,1]$, then $T_t \in [0,1]$ for all t. Under mild

ergodicity conditions on the underlying interaction/recommendation processes, each component converges in expectation to its long-run mean and therefore T_t converges in expectation to a convex combination of these means. Because the weights $w_{j,t}$ are normalized and derived from past trust values, they remain bounded and do not induce divergence.

Definition and normalization of TRF. Recall TRF between nodes v_i and v_j is defined as

$$\mathrm{TRF}_{ij} = \gamma F_{ij} + \delta I_{ij}, \tag{7}$$

where F_{ij} is the interaction frequency (normalized to $[0, 1]$ over a sliding window) and I_{ij} is the importance metric (also normalized to $[0, 1]$). For clarity and stability we recommend imposing $\gamma, \delta \geq 0$ and $\gamma + \delta = 1$; normalization of F_{ij} and I_{ij} should be performed by min–max or by dividing by the maximum observed value over the calibration window.

Principled TRF threshold selection. We recommend a hybrid, reproducible approach comprising three options depending on the availability of labeled calibration data and deployment constraints.

Option A (ROC-based calibration, preferred when labeled calibration data are available). Let τ denote the TRF threshold and define the detection rule "propagate if $\mathrm{TRF}_{ij} > \tau$". Compute true positive rate $\mathrm{TPR}(\tau)$ and false positive rate $\mathrm{FPR}(\tau)$ over a labeled calibration set. Choose

$$\tau^* = \arg\max_{\tau} \big(\mathrm{TPR}(\tau) - \mathrm{FPR}(\tau)\big), \tag{8}$$

i.e., maximize Youden's J statistic to obtain the operating point with the best trade-off between sensitivity and specificity. Alternatively, select τ to satisfy a deployment-specific constraint on FPR.

Option B (statistical adaptive rule, for online operation). Maintain a rolling window of the most recent W TRF samples among relevant neighbors, compute the rolling mean μ_{TRF} and standard deviation σ_{TRF}, and set

$$\tau(t) = \mu_{\mathrm{TRF}}(t) + k\,\sigma_{\mathrm{TRF}}(t), \tag{9}$$

where k is chosen to control the Type I error (e.g., $k = 1.96$ for a one-sided 97.5% threshold under approximate normality). This rule adapts to local traffic density and behavior.

Option C (percentile-based fallback, for deployments without labeled data). Compute the empirical p-th percentile Q_p of TRF over a calibration period and set $\tau = Q_p$ with p in $[0.70, 0.90]$ depending on desired sensitivity.

For typical vehicular network deployments, we recommend the following initial parameter settings: an EWMA smoothing factor $\lambda \in [0.05, 0.25]$, where smaller values yield smoother but slower trust updates; a TRF importance weight $\delta \in [0.5, 0.7]$, with $\delta = 0.6$ used in our baseline experiments to emphasize message criticality; and a calibration window W chosen based on expected neighbor churn. These parameters should subsequently be validated and fine-tuned using real deployment traces or the VeReMi dataset through the ROC-based calibration procedure described above.

"AT the end, we would like to thank the referees and editors for evaluating our manuscript. We have tried to address all the reviewers' concerns in a proper way and believe that our paper has improved considerably. We would be happy to make further corrections if necessary and look forward to hearing from you soon."
