# Supplementary material for: Decentralized trust optimization in VANETs: A blockchain-driven hybrid PoS-PBFT architecture for enhanced security and energy-efficient communication
Source: PLoS One. 2025 Dec 15;20(12):e0338618. doi: 10.1371/journal.pone.0338618 (PMC12704869; doi:10.1371/journal.pone.0338618)
Supplement: Attachment — Submitted filename: PlosOne Rebuttal-III (PONE-D-25-29277R2).docx [file pone.0338618.s003.docx]

**Manuscript ID: PONE-D-25-29277R2**

**Original Article Title:** "Decentralized Trust Optimization in VANETs: A Blockchain-Driven Hybrid PoS-PBFT Architecture for Enhanced Security and Energy-Efficient Communication"

**To:** **PLOS One** Editor

**Re:** Response to Reviewers

Dear Editor,

Thank you for allowing a resubmission of our manuscript, with an opportunity to address the reviewers’ comments. The revised draft of our Manuscript ID **PONE-D-25-29277R2 entitled " Decentralized Trust Optimization in VANETs: A Blockchain-Driven Hybrid PoS-PBFT Architecture for Enhanced Security and Energy-Efficient Communication"** to **PLOS One**. We are thankful to you for spending your precious time in thoroughly reviewing our manuscript in the light of the remarks given by two worthy reviewers. We are uploading (a) our point-by-point response to the comments (below) (response to reviewers), (b) an updated manuscript with yellow highlighting indicating changes (*Supplementary Material for Review*), and (c) a clean updated manuscript without highlights (*Main Manuscript).*

We will be happy to make any further improvements that may be suggested by the reviewers.

Best Regards,

**Zia Ullah et al.**

**Manuscript ID: PONE-D-25-29277R2**

**Journal:** **PLOS One**

**Manuscript Title: "** Decentralized Trust Optimization in VANETs: A Blockchain-Driven Hybrid PoS-PBFT Architecture for Enhanced Security and Energy-Efficient Communication**"**

**Reviewer #1: All comments have been addressed.**

**Comments to the Authors:**

**Reviewer #1: In my opinion, all the previous comments and concerns have been successfully adressed by the authors. So the paper can be accepted in its current form.**

**Reviewer #2: All comments have been addressed.**

**Comments to the Authors:**

Reviewer #2: The manuscript presents a blockchain-based decentralized trust management system for Vehicular Ad Hoc Networks (VANETs), combining Proof of Stake (PoS) and Practical Byzantine Fault Tolerance (PBFT) into a hybrid consensus model. The topic is relevant, and the authors provide a clear motivation for improving scalability, latency, and energy efficiency in trust evaluation for vehicular communication networks.

The overall structure of the paper is coherent, and the methodology is presented with sufficient mathematical and algorithmic detail. The revised version effectively addresses most reviewer concerns. The authors now provide a well-justified explanation of the Trust Authority’s limited supervisory role, add convergence analysis for the EWMA-based trust updates, and include comparative evaluation metrics such as block finality and validator latency. The decision to release cleaned simulation and smart contract code on GitHub also significantly improves the transparency and reproducibility of results.

The implementation corresponds closely to the described system design. The simulation modules correctly include EWMA-based trust updates, TRF computation with ROC-based threshold calibration, dynamic PoS validator selection, and simplified PBFT consensus rounds. However, some elements remain simplified. The PBFT mechanism is modeled at a basic level without realistic communication or geofencing. The mobility model is random rather than map-based, and the energy-efficiency claims rely on indirect metrics. Moreover, the machine learning–based malicious node detection described in the text is not implemented in the released code.
In summary, the paper makes a meaningful contribution to decentralized trust management in VANETs. The work demonstrates a consistent and technically sound integration of blockchain mechanisms with trust dynamics, though parts of the implementation could benefit from further refinement and empirical validation.

The authors are encouraged to,

**Reviewer#2, Concern # 1: (i) clarify the limits of their simulation model,**

**Author Response:**

We thank the reviewer for this valuable comment and for recognizing the technical scope of our simulation framework. We acknowledge that the present study is limited to a controlled simulation scale and does not attempt to capture the full complexity of a large-scale or real-world VANET deployment. The simulation model was intentionally designed for small to medium-scale environments, emphasizing localized trust management zones where vehicles interact within limited communication ranges. The selected configuration, consisting of 100 vehicles distributed over an area of 4 square kilometers, reflects an urban or semi-urban scenario that allows meaningful analysis of trust dynamics, consensus latency, and communication overhead under realistic traffic densities. This setup ensures that experiments remain computationally tractable and reproducible while preserving the integrity of decentralized trust evaluation. Certain elements, such as large-scale topology, heterogeneous road networks, and long-range multi-hop routing, were simplified to maintain experimental control and computational feasibility. Including such complexities would require extensive infrastructure and simulation resources beyond the current scope. However, these simplifications do not diminish the conceptual validity of the proposed model, as the underlying trust evaluation, consensus coordination, and validator selection mechanisms are designed to be scalable and adaptable to broader deployments.

We have now clarified these scope boundaries and assumptions in the revised manuscript to provide a transparent description of the simulation limitations and to guide readers regarding the contexts in which the proposed system is most applicable.

**Author Action:**

We have updated the manuscript according to the reviewer instructions and highlighted the changes with YELLOW color **Section 5.1 Simulation Tools and Environment, 5.2 Simulation Scenario and Parameters, 5.3 Hardware and Software Configuration (Please See on P#23-26)** in the revised manuscript.

**Reviewer#2, Concern # 2: (ii) include a brief discussion of the missing ML component and energy metrics,**

**Author Response:**

We appreciate the respected reviewer for in depth observation regarding the partial implementation of the machine learning component and the use of indirect energy metrics in the current version of the manuscript. The Gaussian Naive Bayes (GNB) model was conceptually integrated within the proposed trust management framework to support malicious node classification based on behavioral features. However, due to computational and synchronization constraints associated with the co-simulation setup between SUMO, OMNeT++, and Hyperledger Fabric, the full end-to-end implementation of the GNB-based classifier was excluded from the released simulation code. Instead, the behavioral data were pre-processed offline to estimate detection accuracy and validate model consistency. This methodological choice allowed us to maintain the fidelity of trust dynamics while avoiding instability in the distributed simulation environment.

Regarding the energy consumption metrics, we acknowledge that direct hardware-level measurements were not feasible within the virtualized simulation environment. Consequently, energy efficiency was inferred from proxy indicators such as consensus latency, message transmission count, and validator workload where each of which correlates strongly with energy consumption in blockchain-based vehicular networks. These indirect measures provide a reliable approximation of relative energy performance, particularly when comparing the proposed hybrid PoS-PBFT model to energy-intensive Proof-of-Work schemes.

Future work will extend the experimental setup to include (i) a fully integrated machine learning module operating in real time within the co-simulation loop, and (ii) detailed energy profiling using realistic vehicular power models or hardware-in-the-loop testing to capture the physical-layer energy implications of trust and consensus operations.

**Author Action:**

We have updated the manuscript according to the reviewer instructions and highlighted the changes in **Section 6 Result and Discussion** for incorporating real-time machine learning integration and hardware-based energy analysis in future work, with YELLOW color in the revised manuscript.

**Reviewer#2, Concern # 3** **(iii) describe more explicitly how the hybrid PoS–PBFT can be extended to large-scale, realistic VANET scenarios.**

**Author Response:**

We sincerely thank the reviewer for this valuable observation regarding the scalability of the proposed hybrid PoS–PBFT consensus mechanism. While we fully acknowledge the importance of validating the framework under large-scale and highly dynamic VANET conditions, this aspect lies beyond the practical and methodological scope of the current study. The proposed system was intentionally designed and evaluated within small- to medium-scale vehicular environments to ensure methodological rigor, reproducibility, and computational feasibility. Large-scale deployments involving thousands of vehicles would require a dedicated simulation and network infrastructure capable of handling complex map-based mobility, multi-hop propagation models, and geographically distributed validator coordination. Incorporating such features in the current setup would not only demand substantial computational resources but would also complicate the isolation of core performance metrics such as trust convergence, consensus stability, and propagation latency which are the central focus of this research.

Our present implementation, therefore, serves as a proof-of-concept validation of the hybrid PoS–PBFT model operational soundness and its advantages in reducing consensus latency, improving trust accuracy, and minimizing communication overhead in moderately dense vehicular networks. The insights gained from these controlled experiments establish a strong foundation for future work focused on large-scale scalability testing.

We have also emphasized in the discussion and conclusion sections that extending the hybrid consensus framework to large-scale, real-world VANET deployments remains an important direction for future research. This will involve incorporating hierarchical consensus layers, dynamic validator clustering, and mobility-aware sharding mechanisms to maintain scalability without compromising fault tolerance or security guarantees.

**Author Action:**

No changes were made to the current version of the manuscript regarding large-scale implementation, as the experimental scope and objectives were clearly defined to validate the core functional behavior of the proposed trust management framework.

*“AT the end, we would like to thank the referees and editors for evaluating our manuscript. We have tried to address all the reviewers’ concerns in a proper way and believe that our paper has improved considerably. We would be happy to make further corrections if necessary and look forward to hearing from you soon.”*
